# The Evaluation of Municipal Waste in Counties in Poland with the Use of the Theory of Phenomena Spatial Concentration

**DOI:** 10.3390/ijerph17239107

**Published:** 2020-12-06

**Authors:** Iwona Krzywnicka, Katarzyna Pawlewicz, Adam Senetra

**Affiliations:** Department of Socio-Economic Geography, University of Warmia and Mazury in Olsztyn, Prawocheńskiego 15, 10-720 Olsztyn, Poland; iwona.krzywnicka@uwm.edu.pl (I.K.); adam.senetra@uwm.edu.pl (A.S.)

**Keywords:** selectively collected waste, mixed waste, concentration of spatial phenomena, location quotient, waste management monitoring

## Abstract

In the era of increased consumption and with the development of new technologies, waste management and its constant monitoring are some of the greatest challenges for humanity. The aim of this article is to analyze and evaluate the condition of mixed municipal waste management and the selectively collected waste in all counties (LAU 1) in Poland. The authors chose six fractions of selectively collected waste. The theory of the concentration of spatial phenomena was selected for the evaluation. The analytical part was based on the concentration coefficient (*CC*) and the location quotient (*LQ*). The created maps of the correctness of waste management allow for formulating corrective actions for the analyzed counties. The fractions of selective waste, whose management is balanced to the greatest extent on the national scale, are those for which the value of *LQs* is on a similar level—glass, biodegradable waste and bulky waste. However, in the case of paper and cardboard, plastic and waste electrical and electronic equipment fractions, vast disproportions in waste management were noted. The proposed methodology can be an effective tool of constant monitoring and for planning the process of waste management on a local, regional and national scale.

## 1. Introduction

Modern consumer society is producing more and more waste. Designing an efficient system of waste management depends on a precise prognostication of the produced amount of waste [1]. Nowadays, recycling is the basis of the modern system of municipal waste management [2], and its selective collection is the best means for achieving the goal [3,4].

Waste is a real threat to the environment also in the context of global warming. It was estimated that in 2016, 1.6 billion tons of carbon dioxide (CO_2_) was produced from the waste sector, representing approximately 5% of global emissions [5,6]. According to the forecasts for the year 2050, the emission will reach 2.6 billion tons of CO_2_ equivalents unless there is an improvement in waste management. Even though waste is 5% of the total CO_2_ emissions, which is lower in comparison with the energetic or transportation sectors, some authors state that people should aim to create intelligent waste management in order to alleviate and adjust to climatic changes. The limitation of the excessive amount of waste and directing it to other options of waste management is a cheap and possible way of decreasing the carbon footprint in organic waste management. Solutions have been observed in developed countries in the European Union, Japan, and so on [6].

The Directive 2008/98/EC [7], which is the key legal act in the European Union in waste management, suggests creating “the recycling society”, which would aim at avoiding waste production and using waste as sources. The document supposed that by 2015, the selective waste collection would apply at least to paper, metal, plastic and glass. Moreover, it assumed that until 2020, the preparation for reusing and recycling waste material (paper, metal, plastic and glass coming from households and as far as possible from other sources, on the condition that the streams of waste are similar to those coming from households) in terms of weight would be increased to a minimum of 50%. The change in the directive introduced on the 30th of May 2018 [8] additionally encouraged countries to save food (collecting unsold food products, food donations and other forms of redistribution), which consequently should contribute to reducing organic waste. Additionally, the aims regarding the increase in the preparation of waste for reusing and recycling to 55% in 2025, to 60% in the year 2030 and to 65% in 2025, were included. Moreover, the possibility of changing the dates (under justified circumstances) to 5 years with the decrease in factors to 50% in 2025, 55% in the year 2030 and 60% in 2035 were determined. In order to control the assumed effects, the unification of rules for estimating the degree to which the aims would be achieved for all member countries were proposed.

In Poland, the percentage of municipal waste directed to recycling was 26.73% (in 2017) and 26.18% (in 2018). Moreover, the collected municipal waste destined to compost and fermentation was 7.08% (in 2017) and 8.11% (in 2018), and the waste for thermal processing with the energy recover was 22.76% (in 2017) and 22.60% (in 2018) [9].

Data of the Central Statistical Office, quoted in the National Waste Management Plan 2022, show that in Poland, the percentage of stored municipal waste had decreased from 94.21% in 2004 to 52.63% in 2014 [10]. According to the data of the Central Statistical Office, in 2017 the percentage was 41.77%, and in 2018, 41.58%.

The directive of the Environment Minister on the detailed ways of the selective collection of given waste fractions [11] determines the fractions of selective collection: (1) paper; (2) glass; (3) metal; (4) plastic; and (5) biodegradable waste, with special regard to bio-waste. These are fractions for which selective collection is obligatory for all inhabitants. The obligation was introduced in 2019. In Poland, municipalities are responsible for keeping the areas clean and in order, i.e., they have to create points for selective collection of municipal waste in such a way that the points are easily available for all inhabitants of a county. The points must take municipal waste, such as paper, metal, plastic, glass, packaging waste, multi-material waste and bio-waste, hazardous waste, used batteries and storage batteries, used electric and electronic equipment, furniture and other bulky waste, used tires, construction and demolition waste and textile and clothing waste from all estates [12].

For selecting the fractions, the following aspects were considered:

1. Legal regulations [11,12,13,14,15].
2. Determining a draft list of suggested fractions on the basis of a literature review, especially Polish positions [2,4,16,17,18,19,20,21], which were verified considering the data availability and their completeness for all the objects.
3. The highest share of a fraction in total selective waste mass [9]. For the analysis, the authors chose only the fractions that have a large weight share of selective waste and rejected those fractions that are rarely collected or do not even appear in given counties. Due to the lack of data, the fractions were not included in the research, and the authors selected those with 99% completeness of data.

The aim of this work is to evaluate the management of mixed municipal waste and selectively collected waste (in the following text related to as mixed and selected waste) for all counties in Poland. Moreover, the selective waste collections of particular fractions were compared in all counties in Poland (380) with the use of the spatial concentration coefficient (*CC*) and the location quotient (*LQ*). The coefficients can be useful tools in the process of spatial planning. The fractions selected for the analyses include paper and cardboard (*PC*), glass (*G*), plastic (*P*), waste electrical and electronic equipment (*WEEE*), bulky waste (*BW*) and biodegradable waste (*B*).

### Methods for Evaluating Municipal Waste Management

The increased production of waste, especially municipal waste, and ineffective use of natural resources still influence the environment and change it [22]. This problem has become one of the most important challenges for modern civilization, and the produced waste requires particular management by its neutralization and reuse. Unfortunately, one of the most frequent ways of waste utilization is its disposal at landfills. Consequently, the growing number of landfills contributes to environmental degradation by contaminating soils, waters, the atmosphere; by the deterioration of landscape; and by becoming a local epidemiological threat [4]. Therefore, we must be aware of such threats to the environment and try to minimalize the negative consequences to the largest possible extent for the wealth and health of future generations [22]. Waste treatment can contribute to reaching this aim. Between 1995 and 2016, the efficiency and convergence in recycling municipal waste in 27 European Union countries were evaluated by Giménez et al. [23]. The highest results were gained by countries in Central and Northern Europe (Germany, Austria and Denmark), and the lowest results were achieved in the eastern part of Europe, that is, in countries that joined the EU after the year 2000.

Recycling is one of the possibilities of waste treatment. Recycling is in the line with the idea of sustainable development, according to which waste should be reused, i.e., recycled. Introducing and applying recycling is possible with the use of methods for waste selective collection and recovery [4]. The increasing social awareness of the pollution of the environment influences the reduction of pollution. However, the amount of municipal waste has been increasing since 2011 [9]. Thus, a selective collection of given fractions can be effective, as it allows the use of waste as a source. In this context, in order to draw out the recommendations for fulfilling the required factors, one must evaluate the selective waste collection, considering particular waste fractions. Additionally, the possibility of comparing the activities in various administrative units and on various administrative levels can be an added benefit.

The evaluation of municipal waste management conducted by administrative units depends to a large extent on the effectiveness of waste collection and, nowadays, also on the effectiveness of selective waste collection. The important role here is a thorough information–education campaign which can raise ecological awareness [24]. De Feo et al. [25] came to similar conclusions, as they suggested a methodological approach in order to define indicators that can be implemented in communication campaigns organized to improve the efficacy of municipal waste collection. The authors determined six socio-economic indicators. The methodology was applied for paper and cardboard collection in twelve cities in southern Italy. The main assumption of the innovative method of communication (“Greenopoli”) used in this case states the starting point for changes in the attitude is in schools, as it gives indirect access to potential and the most important recipients (addressees) of communication campaigns [25]. In Polish conditions, the authors Lorek [26], Deluga [17] and Pawlewicz et al. [19] also draw attention to the meaning of educational and pro-ecological activities by analyzing the principles of waste management on the basis of a questionnaire survey conducted among the inhabitants of research administrative units. Furthermore, an effective selective waste collection is connected with a proper infrastructure, which was then indicated by Wengierek [18] by evaluating the condition of municipal waste management in cities in the Silesian region. As a result of the quantity description method which she applied, it was possible to conduct analyses that became the bases for the statement that the level of recycling of selectively collected waste, biodegradable waste, construction and demolition waste between 2012 and 2013 depended on the facilities located in given cities and was higher in comparison to cities deprived of such facilities. For example, in cities that had composting plants, the level of selectively collected waste was 100% (Ruda Śląska). Due to the construction and demolition of a waste sorting plant, the salvage from selectively collected waste was 100% (Zabrze, Tarnowskie Góry, Chorzów).

According to Polish legislation [12], maintaining order and cleanliness in municipalities is the duty of the municipalities. As Lewandowska and Szymańska [21] showed in their research, the problems with selective waste collection stem from the divergence between the attitudes of city governments to waste management. According to them, in places where the local government strives for the ecologization of space, it is easier to implement the regulations on sustainable waste management. The document which allows for ordering the activities of local governments connected with waste management in administered areas is the Waste Management Plan. The effectiveness of the instrument was analyzed by Alankiewicz [16], who suggested a universal segmentation evaluation method, which main aim was to evaluate the realization of the Municipal Waste Management Plan. The indicators created by the author can be applied for evaluating the management of mixed and selective municipal waste not only on the level of municipalities.

According to the data of The Ministry of Environment, the most often segregated waste types in Poland are plastic, glass and paper [24]; however, as shown in the research conducted by Senetra et al. [20], who analyzed the management of selected and mixed municipal waste for voivodeship cities in Poland, the greatest discrepancies appear in collecting the fractions on the level of the analyzed cities. However, Cole et al. [27] indicated that waste electronic and electric equipment is one of the fastest-growing amounts of waste in the European Union. According to the authors, regardless of the determined “waste hierarchy”, which states that waste prevention and re-usage is better than recycling, recycling remains dominant in e-waste treatment. As proved in the research conducted in Great Britain, re-usage occurred in a limited range, and recycling was limited to the easiest ways of salvaging resources. To increase the level of salvaging resources, a range of means was proposed: the promotion of such practices which facilitate re-using given products and adjusting the recycling technology to increase the amount of regained critical raw materials.

There are not any methods for managing municipal waste as a whole, which causes a lack of coordinated activities for all spatial units. The activities are mainly limited to solving the problems at the administrative units’ level and facing problems with particular waste fractions. It is essential to take action in order to create rules for managing municipal waste on a larger scale, which would then contribute to increasing the effectiveness of pollution prevention. The numerous ways to evaluate municipal waste management presented in this Section concern research conducted on various administrative levels and also by using various indicators for evaluating different fractions. Each of them is useful in the range it was used. Comparing the effectiveness of the methods is difficult due to the above-mentioned reasons.

## 2. Materials and Methods

**Figure 1:**
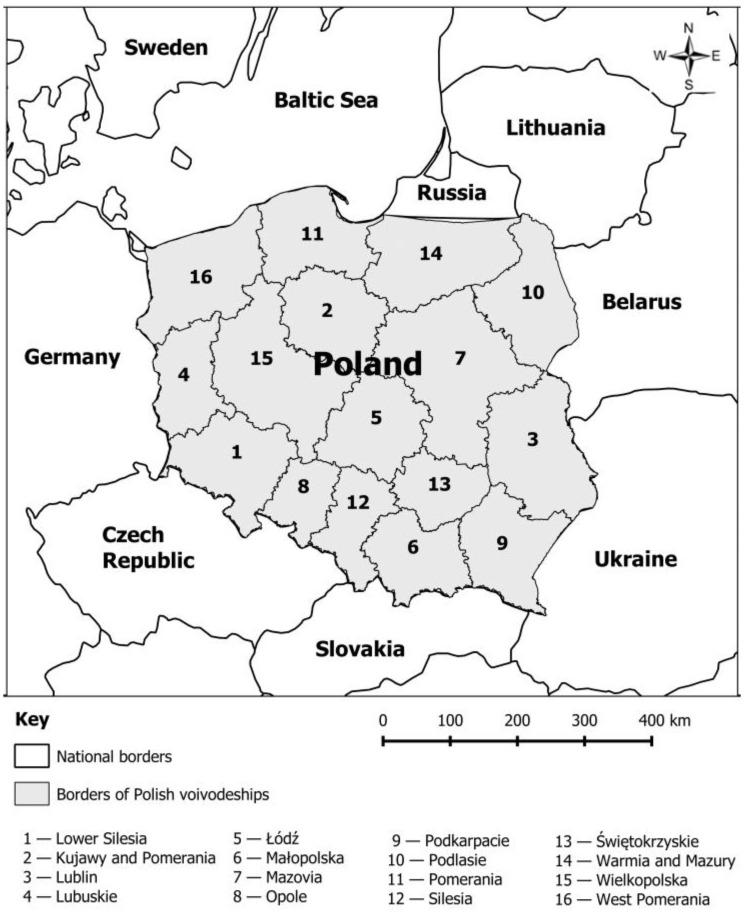
The location of the research object. Source: own elaboration.

This research was conducted on the administratively divided counties of Poland. Statistical data regarding selected waste with the division in fractions were taken from the Central Statistical Office in Municipal Office (LAU 2, formerly NUTS 5). Previously, data were provided only with the division into voivodeships (NUTS 2). Due to a large number of counties in Poland, which profoundly hindered cartographic and tabular representation of the obtained results, it was decided to choose the counties (LAU 1, formerly NUTS 4) as the analyzed units. The numbers of voivodeships presented in Figure 1 correspond to the order of records in the Local Data Bank in the Central Statistical Office.

Poland is located in central Europe (Figure 1). It borders with Lithuania and Russia in the north (Kaliningrad Oblast), and with Belarus and Ukraine in the east. These, excluding the Lithuanian border, also represent the European Union borders. In the south, Poland borders with Slovakia and the Czech Republic and in the west, with Germany. The majority of the northern border is the coastline of the Baltic Sea. The administrative surface of Poland is 312,695 km^2^. The population of Poland is 38,411 million (2018) [9]. The country is divided into 16 voivodeships, which are also divided into 380 counties (314 county districts and 66 cities with a county status). For statistical purposes, they are LAU 1 levels. Counties are the subordinate units. There are 2478 municipalities in Poland.

For the evaluation of the condition of municipal waste management in counties in Poland, the theory of phenomena spatial concentration and the location quotient were selected. In this way, the following issues were evaluated:

1. The level of selectively collected municipal waste in comparison with the total municipal waste.
2. The level of the six fractions of selectively collected municipal waste in comparison with the total selectively collected waste.

All data concerning mixed and selected waste in all counties in 2018 were gained from the Local Data Bank of the Central Statistical Office [9]. The authors analyzed 380 counties.

The research on the phenomena spatial distribution consists of determining the degree of concentration of the analyzed phenomenon in comparison with another phenomenon. The comparison shows the degree to which the concentration of the research phenomenon deviates from the concentration of the basic phenomenon.

The concentration coefficient suggested by Florence is presented as follows [28,29]:

(1) $$CC=\frac{\sum_{i=1}^{n}\left(x_{i}-y_{i}\right)}{100}$$

where:

*CC*—concentration coefficient;
*x_i_*—the percentage share of the *i* unit in the global value of the research phenomenon;
*y_i_*—the percentage share of the *i* unit in the global value of the basic phenomenon;
*n*—the number of individual units.

Note: the sum of positive or negative differences is calculated (the sum of positive differences and the sum of negative differences have the same absolute value).

**Figure 2:**
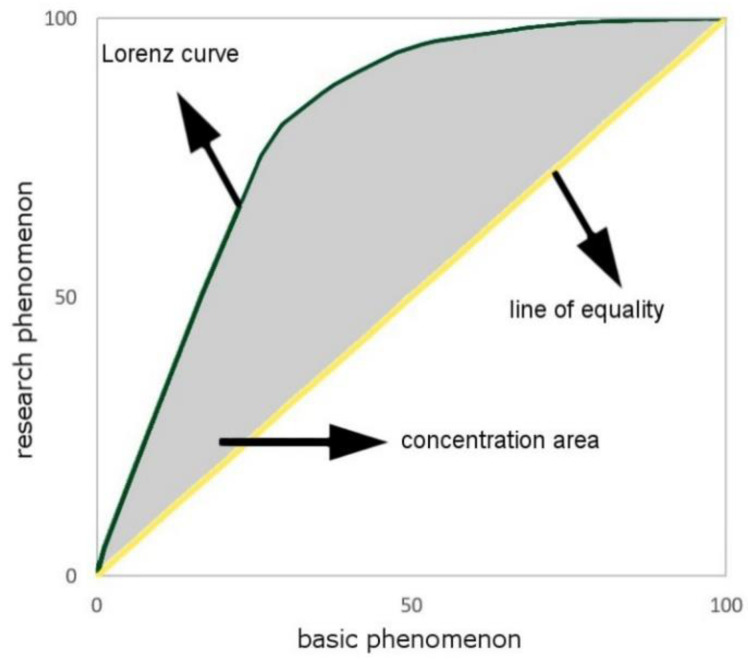
Lorenz curve. Source: own elaboration.

The coefficient *CC*, which consists of the range <0, 1>, is the unit-less measure. It reaches 0 in the case of a full dispersion—when the research phenomenon is distributed as the basic phenomenon. Otherwise (a full concentration), the coefficient reaches value 1. This means that the research area is concentrated on one unit field [30,31]. The Lorenz curve is the graphic representation of the *CC*. In the case of the even distribution of the research phenomenon, the diagram is a straight line starting at the beginning of the coordinate system, which is inclined at the angle of 45°. Each inclination from the proportion forms the curve convex (Figure 2). In the beginning, the diagram is created by putting the units in order, according to the decreasing value of the *LQ* (the quotient of the percentage share of the research phenomenon and the percentage share of the basic phenomenon). In the subsequent stage, the percentage values agreed for each range are cumulated—in accordance with the order designated by the *LQ*. The cumulated range of the research phenomenon is then put on the vertical axis, and the cumulated range of the basic phenomenon is marked on the horizontal axis [28,31]. The surface between the straight line inclined at the angle of 45° and the Lorenz curve is the interpretation and graphical measurement of the concentration. The relation of the surface to the total surface of the upper triangle equals the *CC* (Figure 2).

In order to evaluate the level of selectively collected waste in the total waste mass (mixed and selected) in particular counties, data regarding general municipal waste and the following six fractions were used:

1. Paper and cardboard (*PC*).
2. Glass (*G*).
3. Plastic (*P*).
4. Waste electrical and electronic equipment (*WEEE*).
5. Bulky waste (*BW*).
6. Biodegradable waste (*B*).

The justification for choosing the fractions is their highest share in the mass of selected waste, as well as the fact that they are the most common selectively collected waste. First, the concentration coefficient of all selected waste (*CCsw*) in the research counties was calculated (Formulas (2)–(4)). The basic phenomenon (*BPH*) was described as the quotient of the sum of mixed waste and selected waste in every county, and the sum of the waste in all research counties (Formula (2)). The research phenomenon (*RPH*) was determined as the quotient of the amount of selected waste in every county and the sum of selected waste in all counties (Formula (3)).

(2) $$BPH=\frac{MSW\;in\;county\;i}{MSW\;in\;all\;counties}$$

where:

*MSW*—the mass of mixed and selected waste all together.

(3) $$RPH=\frac{SW\;in\;county\;i}{SW\;in\;all\;counties}$$

where:

*SW*—the mass of selected waste.

The concentration coefficient of all selected waste (calculated with Formula (1)) takes the following form (Formula (4)):

(4) $$CCsw=\frac{\sum_{i=1}^{380}\left(RPH-BPH\right)}{100}$$

where:

$\overset{380}{\underset{i=1}{\sum }}\left(RPH-BPH\right)$—the sum of the absolute difference of the research phenomenon and the basic phenomenon. Note: the sum of positive or negative differences is calculated (the sum of positive differences and the sum of negative differences have always the same absolute value).

Similarly, the degree of the concentration of selectively collected waste with the division into six fractions was estimated. The basic phenomenon (*BPH*) in the case of all fractions is the value of the research phenomenon (*RPH*) in Formula (3). Then, the research phenomena (*RPH*) for all waste fractions were defined with the following formulas:

1. Selected waste, “paper and cardboard”—*PC*:
  (5) $$RPH_{PC}=\frac{SW_{PC}\;in\;county\;i}{SW_{PC}\;in\;all\;counties}$$
  where:
  *SW_PC_*—the mass of selected waste *PC*.
2. Selected waste, “glass”—*G*:
  (6) $$RPH_{G}=\frac{SW_{G}\;in\;county\;i}{SW_{G}\;in\;all\;counties}$$
  where:
  *SW_G_*—the mass of selected waste *G*.
3. Selected waste, “plastic”—*P*:
  (7) $$RPH_{P}=\frac{SW_{P}\;in\;county\;i}{SW_{P}\;in\;all\;counties}$$
  where:
  *SW_P_*—the mass of selected waste *P*.
4. Selected waste, “waste electrical and electronic equipment”—*WEEE*:
  (8) $$RPH_{WEEE}=\frac{SW_{WEEE}\;in\;county\;i}{SW_{WEEE}\;in\;all\;counties}$$
  where:
  *SW_WEEE_*—the mass of selected waste *WEEE*.
5. Selected waste, “bulky waste”—*BW*:
  (9) $$RPH_{BW}=\frac{SW_{BW}\;in\;county\;i}{SW_{BW}\;in\;all\;counties}$$
  where:
  *SW_BW_*—the mass of selected waste *BW*.
6. Selected waste, “biodegradable waste”—*B*:
  (10) $$RPH_{B}=\frac{SW_{B}\;in\;county\;i}{SW_{B}\;in\;all\;counties}$$
  where:
  *SW_B_*—the mass of selected waste *B*.

The transitional stage for estimating the concentration coefficient was estimating the location quotient for each county according to the following formula:

(11) $$LQ=\;\frac{BPH}{RPH}$$

The location quotient (*LQ*) is the ratio of the basic phenomenon to the value of the research phenomenon. Additionally, the research results were visualized by cartograms that illustrate the level of waste management with the use of the *LQ*.

The diagrams (together with calculations) of the concentration showed the general trend of the whole research collectivity based on the *LQs* for the objects. However, the precise interpretation of the results for particular units was considerably hindered due to its large number (380). The units characterized with a high *LQ* proved to have a higher level of waste segregation. Therefore, these places had better waste management. The research units (counties) were classified on cartograms into five categories of correct waste management: I the highest, II—high, III—average, IV—low, V—the lowest. The categorization was made with the use of the Jenks natural breaks classification [32]. In this method, it is possible to group similar values and maximize the differences between them. In the research, tools for classifying with the use of the method of natural breaks available in ArcGIS software were used. The method is useful in the case of data with uneven distribution. It is used inter alia for visualizing large sets of data in environmental and socio-economic research [33,34,35]. The analyzed data are characterized by uneven distribution. In order to improve the clarity and a detailed results analysis, the manuscript is complemented with Supplementary Materials Figure S1 of maps of counties in all 16 voivodeships in Poland and tables with the calculation results. Due to the large number of counties, the authors used numbering starting from the first voivodeship given in the Local Data Bank of the Central Statistical Office. Counties in Lower Silesia Voivodeship (the first on the list of the Local Data Bank) start the numbering with 1. Bolesławiecki, 2. Dzierżoniowski, …, 30. c. Wałbrzych. This was subsequently followed by Kujawy and Pomerania Voivodeship (the second on the list by the Local Data Bank) with the numbering of counties: 31. Aleksandrowski, …, 53. c. Włocławek, etc.

## 3. Results

### 3.1. The Concentration of Selectively Collected Waste in Comparison with All Municipal Waste

**Figure 3:**
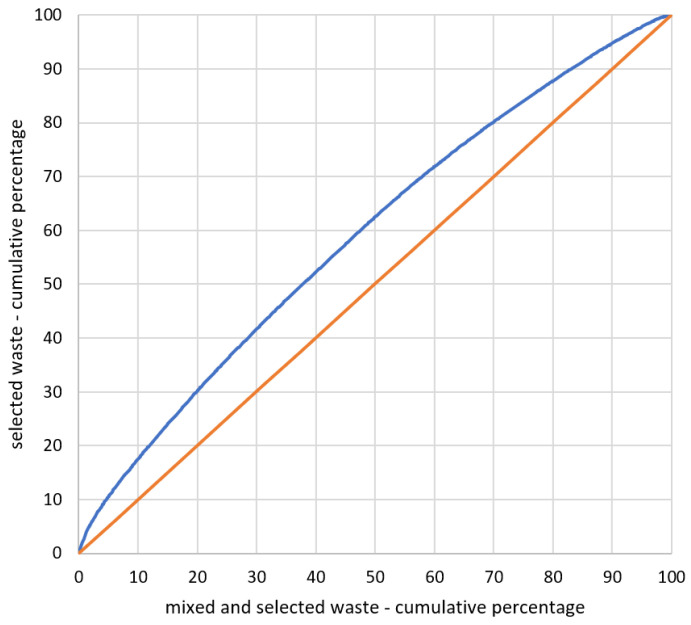
The curve of the spatial concentration of selected waste in comparison with all municipal waste in all counties in Poland. Source: own elaboration.

For the analysis, data collected in the Local Data Bank of the Central Statistical Office [9] concerning municipal waste (both mixed and selected) for 2018 were used. Similar data were prepared for the calculation of the *CCsw*. With the use of the Formulas (2) and (3), the sizes of the basic phenomenon and the research phenomenon for all counties were determined. Then, with the use of Formula (4), the *CCsw* for selectively collected waste in comparison with all municipal waste (both mixed and selected) was calculated. The transitional step in calculating the *CCsw* was determining the *LQ* for each county (Formula (11)). On the basis of the calculated values of *LQs*, the curve of the spatial concentration of selected waste in comparison with all municipal waste in counties in Poland was created (Figure 3). The estimated coefficient was *CCsw* = 0.125.

**Figure 4:**
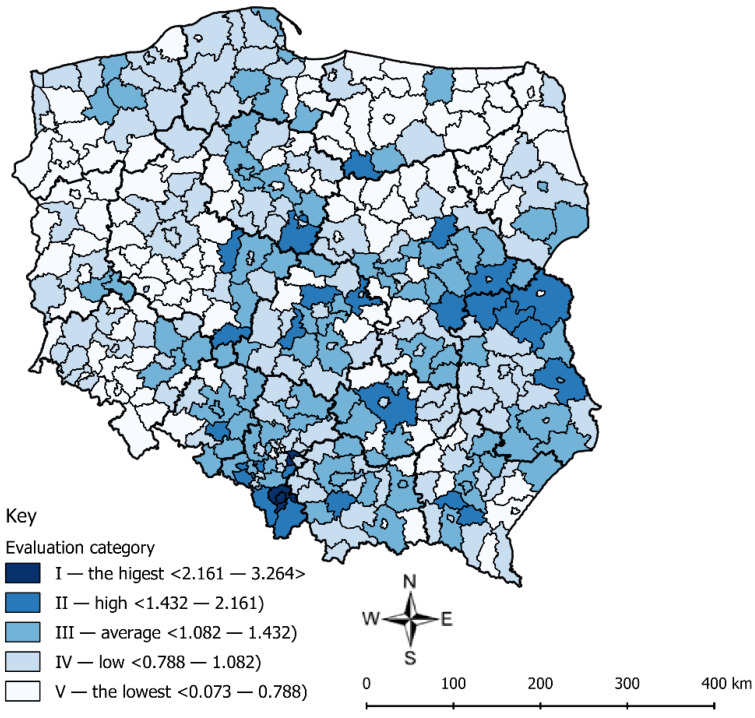
The ranges of the categories of the correctness of waste management for selected waste in comparison with all municipal waste in counties in Poland. Source: own elaboration.

Figure 4 presents the categories of correct waste management determined on the basis of the *LQs* for selected waste in comparison with all municipal waste in counties in Poland.

### 3.2. The Concentration of the Chosen Fractions of Selectively Collected Waste

**Figure 5:**
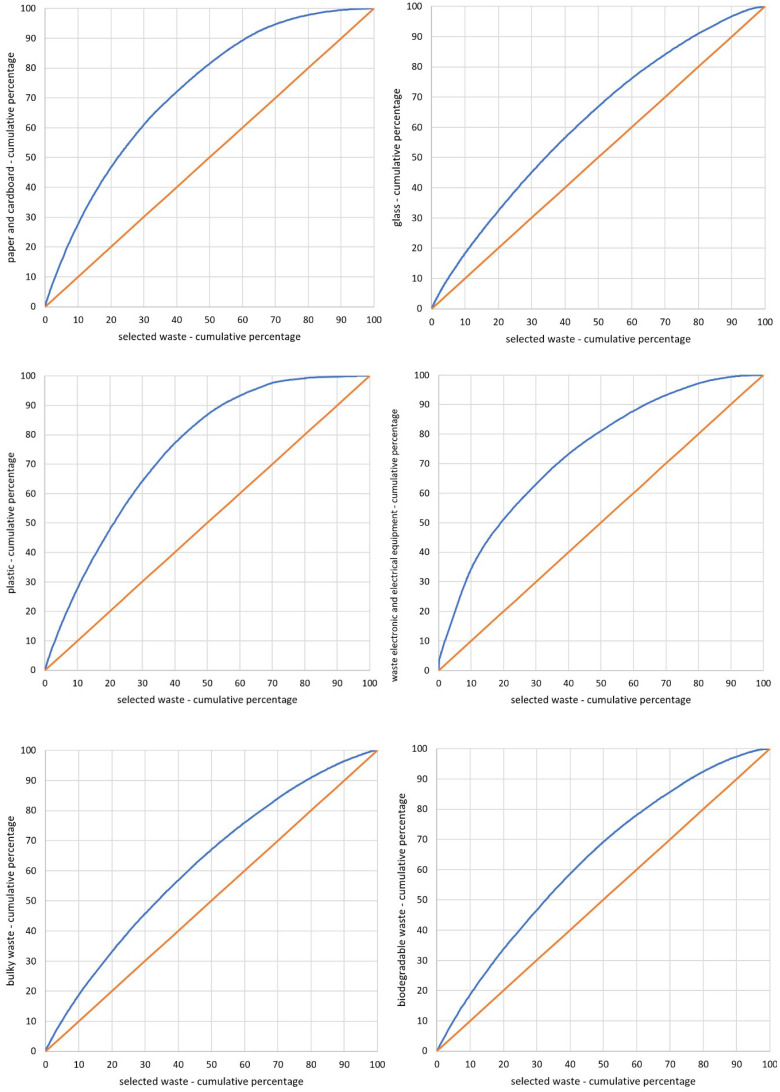
The curves of spatial concentration of the analyzed fractions of selectively collected waste in comparison with all selectively collected waste in counties. Source: own elaboration.

In this case, the basic phenomenon (*BPH*) for all selected fractions is the *RPH* value used in Formula (3). However, the research phenomena *RPH_PC_*, *RPH_G_*, *RPH_P_*, *RPH_WEEE_*, *RPH_BW_*, and *RPH_B_* for particular fractions were estimated using Formulas (5)–(10). The transition phase for calculating each of six concentration coefficients (*CCs*) was estimating the location quotient (*LQ*) for all counties. On this basis, cartograms that present the evaluation of a selective waste collection of given fractions of municipal waste were made. The estimated *CCs* of the given fractions of selected waste are presented in Table 1. The graphic representation of the *CCs* for the analyzed fractions is shown in Figure 5.

**Figure 6:**
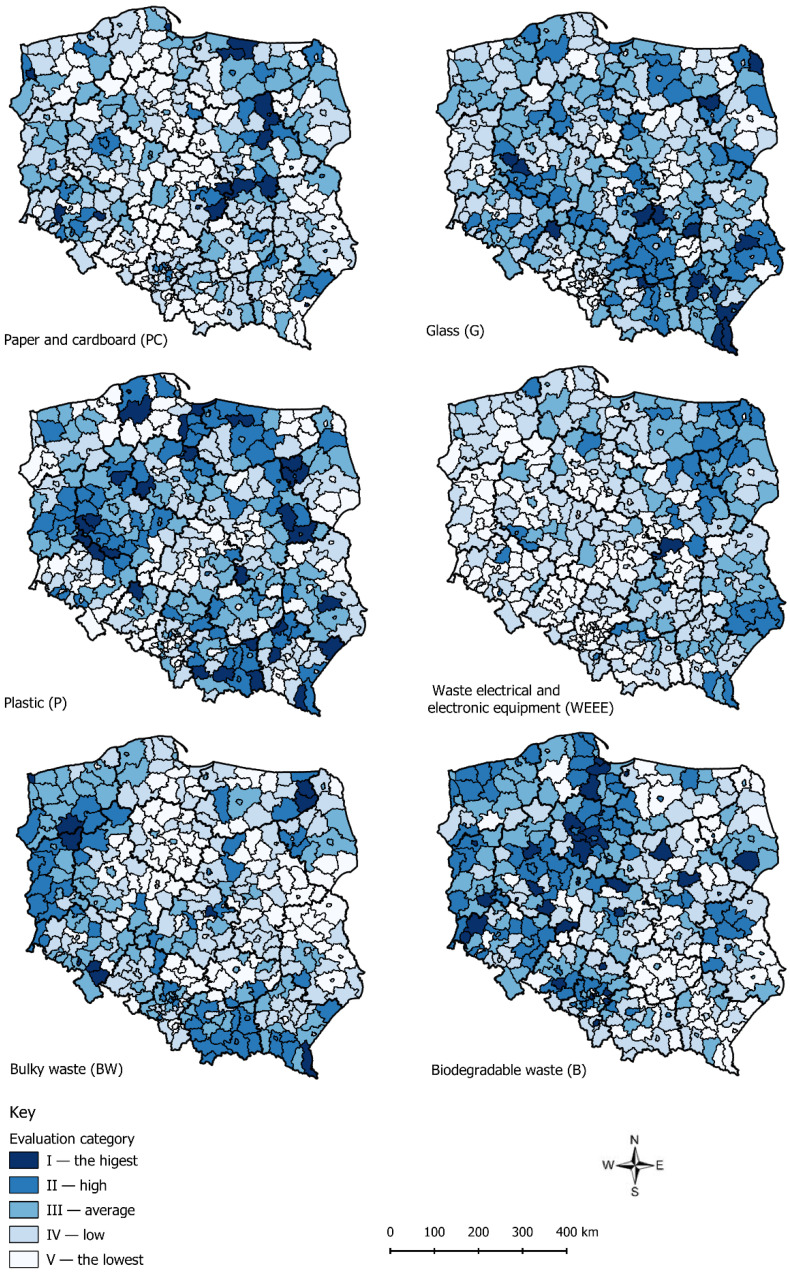
The range of the categories of correct waste management for given fractions of selectively collected waste in counties in Poland. Source: own elaboration.

Figure 6 is the visual representation of the categories of correct waste management for the analyzed fractions of selectively collected waste. The cartograms were based on the *LQs*.

The limits of the ranges of given categories, determined with the use of the natural breaks method, are presented in Table 2.

## 4. Discussion

### 4.1. General Evaluation of Municipal Waste Management

A low coefficient of *CC_SW_* = 0.125 proved that there were no units where waste management was substantially incorrect. The concentration curve slightly deviated from the straight line, which means that the research phenomenon (the relation of the mass of the selected waste in the county to the sum of the mass of the waste in all counties) was similarly concentrated to the basic phenomenon (the relation of the mass of mixed waste and selected waste in the county to the sum of the mass of the waste in all counties). The highest concentration occurred in units where the *LQ* was the highest. When *LQ* ˃ 1, it means that there was the “surplus” of the research phenomenon to the basic phenomenon; when *LQ* = 1, there was a relative balance between the research phenomenon and the basic phenomenon. The quotient *LQ* can be a convenient tool to evaluate a situation in all units in comparison with all analyzed units. The calculated *LQs* for counties remained in the range of 0.073 to 3.264. A small range of the value means that the majority of counties were characterized by a similar level of selective waste collection in relation to the total municipal waste collection.

Based on the research, it can be stated that the highest *LQ* occurred in only four counties: 285. c. Sosnowiec (3.264), 280. c. Mysłowice (3.077), 271. c. Bielsko-Biała (2.894), and 255. Bielski (2.161). It is understood that in those units, the selective collection of waste was on the highest level in comparison with the remaining counties. The counties are marked with the darkest color in Figure 4 (the evaluation category I—the highest). In category II—high, there were 25 counties. A relatively high *LQ* denotes a higher mass of selected waste collected in a given unit in comparison with all units than the mass of selected and mixed waste in the unit in comparison with all units. Next, category III included 90 counties, and the lowest categories (categories IV and V) consisted of 134 and 127 counties, respectively. This means that in these counties, there were irregularities in selectively collected waste management. The lowest *LQ* occurred in inter alia: 30. c. Wałbrzych (0.073), 15. Oławski (0.391), 316. Olecki (0.229), and 321. Gołdapski (0.259). They are marked in Figure 4 with white color (the evaluation category V—the lowest).

### 4.2. Evaluation of Waste Management of the Selected Fractions

The estimated quotients *LQ* for each fraction and all counties allowed for grouping data into categories of waste management correctness. The number of counties for each category is presented in Table 3:

When evaluating the analyzed fractions of selected waste, it should be stated that the *CCs* were the highest for plastic fraction—*CC_P_* = 0.373 (Figure 5 and Table 1). This means that for this fraction, there were units in which waste management was incorrect. The value range of *LQs* for this fraction was between 0.000 and 4.547. The best counties from category I (the highest) were those for which the *LQ* was the highest: 222. Kolneński (4.547), 119. Dąbrowski (4.041), 234. Bytowski (3.985), 34. Górowski (3.909), and 212. Leski (3.930). In general, the category consisted of 30 counties. This fact proved that waste management in numerous counties was generally correct, which is therefore reflected on the map of spatial distribution of the selected categories (Figure 6). The lowest *LQ*, which indicated incorrect waste management, was estimated in the following counties: 332. Kępiński (0.000), 275. c. Dąbrowa Górnicza (0.000), 273. c. Chorzów (0.000), 214. c. Przemyśl (0.000), 184. Krapkowicki (0.000), and 322. Węgorzewski (0.001).

Another fraction marked with a high *CC* was waste electronic and electric equipment—*CC_WEEE_* = 0.334 (Figure 5 and Table 1). The range of this *LQ* was the highest (from 0.000 to 18.059), and it included three counties in which waste management was incorrect. In category I, there were only two units: 143. Grójecki (18.059) and 30. c. Wałbrzych (8.687). The lowest *LQ* was in the following counties: 275. c. Dąbrowa Górnicza (0.000), 185. Namysłowski (0.000), 332. Kępiński (0.001), 177. c. Radom (0.001), and 109. Wieruszowski (0.003). On the map (Figure 6), there is a clear cluster of counties with higher *LQs* (categories I, II and III) located in the east and north-east parts of Poland. In contrast, the counties included in categories IV and V were concentrated in the south and west parts of Poland.

The coefficient *CC* for those two fractions was similar. However, the spatial distribution of particular values of the categories of selected waste management correctness, shown in Figure 6, was significantly different. This is connected with the minimal and maximal values of *LQs*. In the case of *WEEE*, more counties were classified in the IV and V categories, and in the case of *P*, there were not so many differences in the number ranges. Thus, regardless of similar *CC*, the spatial distribution of the numbering of particular categories varied.

Similarly, a high coefficient *CC* was estimated for the paper and cardboard fraction (*CC_PC_* = 0.321). The location quotients were included in the range of 0.000–4.572. In this case, the following counties were in category I: 143. Grójecki (4.572), 140. Ciechanowski (4.416), 26. Złotoryjski (3.600), 379. C. Szczecin (3.369), and 104. Rawski (3.155). The lowest results in this fraction (category V) were noted in the following counties: 332. Kępiński (0.000), 109. Wieruszowski (0.000), 285. c. Sosnowiec (0.009), 38. Lipnowski (0.012), and 292. Kazimierski (0.018). The spatial distribution of the counties with the division into particular categories (Figure 6) shows the cluster of the best (categories I and II) in east-central Poland. In the central part of Poland, which is longitudinal, the cluster of counties with the lowest categories can be observed (IV and V).

Next, the authors analyzed the coefficient *CC* of the biodegradable waste fraction—*CC_B_* = 0.192. The range of *LQs* for counties was between 0000 and 2.333. There were 33 counties in the highest category (category I). The most correct waste management occurred in inter alia 53. c. Włocławek (2.333), 357. c. Konin (2.080), 37. Inowrocławski (2.034), 250. c. Gdańsk (2.009) and 274. c. Częstochowa (2.008). The lowest *LQs*, which indicated incorrect waste management, were seen in the following counties: 160. Przysuski (0.000), 297. Pińczowski (0.005), 110. Zduńskowolski (0.012), 292. Kazimierski (0.013) and 295. Opatowski (0.026). On the map of the categories of correct waste management (Figure 6), a cluster of I, II and III categories is seen in the western part of Poland, whereas counties placed in IV and V categories are located in eastern Poland.

The coefficient *CC* for the bulky waste fraction—*CC_BW_* = 0.171—and the values of the *LQs* varied from 0.005 to 3.411. Category I included the following counties: 30. c. Wałbrzych (3.411), 316. Olecki (2.923), 308. Ełcki (2.864), 24. Ząbkowicki (2.838) and 112. Brzeziński (2.667). The spatial distribution of the phenomenon presented on the map showed that there was a cluster of the counties of the highest categories in the south, north-west, north-east and the central parts of Poland.

The lowest *CC* was noted for the glass fraction (*CC_G_* = 0.169). The quotients *LQs* fluctuated in the range of 0.039–2.923. In category I, there were the following counties: 129. Proszowicki (2.923), 197. Kolbuszowski (2.915), 329. Grodziski (2.718), 212. Leski (2.698) and 160. Przysuski (2.588). The lowest category (V category), included the following counties: 316. Olecki (0.039), 308. Ełcki (0.048), 30. c. Wałbrzych (0.084), 150. Mławski (0.125) and 113. c. Łódź (0.171).

Relatively low *CCs* for the fractions (glass, bulky and biodegradable waste) as well as small differences in *LQs* indicated a similar level of waste collection of the fractions in all of the analyzed counties. When the *LQ* equals 0, the given fraction is not collected in the given county.

The analysis of the *LQs*, the *CCs* and the diagram of spatial concentration of particular fractions of selected waste in comparison with all waste selectively collected showed that none of the fractions reached a *CC* similar to that of selected waste in comparison with all waste (both mixed and selected), which was *CC_SW_* = 0.125. The highest *CC* occurred in the plastic fraction (*CC_P_* = 0.373), which means that the research phenomenon was concentrated in a lower number of the analyzed counties. The concentration curve proves this point, as it significantly deviates from the 45^o^ starting point at the beginning of the coordinate system (Figure 5). The estimated *LQs* for the fraction reached values in the range of 0.000–4.547. A high *LQ* reflected a great concentration of the research phenomenon (plastic selective collection) in comparison with the basic phenomenon (the relation of all of the selected waste in a given county to the amount of selected waste in all counties). This means that in such a county, selective collection of a given fraction remained at a high level. The coefficient *CC*, which was the closest to *CC_SW_*, was the glass fraction (*CC_G_* = 0.169); thus, only this fraction is similarly concentrated like the basic phenomenon.

## 5. Conclusions

The implemented theory of spatial phenomena can make a valuable, clear and effective way of evaluating municipal waste management at the national level. In the elaboration, the evaluation for counties was made. The method can be used at all administrative levels, which had been previously confirmed [20]. The method differs from that described in Section 1 because it recognizes the issue as a whole, and, therefore, it can be used in waste management and not only for the evaluation of selected segments of waste management.

The evaluation of the selective waste collection in comparison with all municipal waste was conducted with the use of the concentration of spatial phenomena and the concentration coefficient (CC). As such, it was possible to identify whether there were counties among those analyzed that substantially diverged from the level in other counties. Therefore, the coefficient *CC_SW_* = 0.125 showed that there were not many counties that differed from the general level of the management of selectively collected waste. The additional information is the value of the LQs, which exceeded the value of 1 in 164 counties, proving that the level of the selected waste collection was high (Supplementary Materials, Table S1).

The evaluation of the selected waste management, which is divided into six fractions, showed that for the three analyzed fractions (glass, bulky waste and biodegradable waste), the *CC* was very similar (Supplementary Materials, Table S2). However, this did not correspond to the spatial distribution of the valorization ranges of the fractions selected with the use of Jenks natural breaks classification. For the remaining fractions (plastic, electrical and electronic equipment, paper and cardboard), the CCs had higher values, which means that there were counties that substantially exceeded the average level.

The coefficients and the diagrams of concentration and the values of LQs are not only useful and valuable tools for evaluating the condition of waste management, but they can also be valuable guidelines for determining the corrective actions for particular spatial units. Moreover, they can also be tools for constant monitoring and planning of the process of waste management on local, regional and national scales. A low LQ in comparison with other units shows that there are irregularities and shows the necessity of the intensification of corrective actions in a given field. The coefficient can be treated as a ranking for the whole system without making further calculations. Its deviations provide information regarding the changes in the level of the phenomenon in the following reporting periods. Furthermore, it shows whether corrective actions should be undertaken or not. Based on the observations of LQs for the whole system, one can also draw conclusions about the accuracy and efficiency of the taken actions.

## Figures and Tables

**Table 1:** The set of the concentration coefficients (*CC*) for the analyzed selected waste fractions.

*CC_PC_*	*CC_G_*	*CC_P_*	*CC_WEEE_*	*CC_BW_*	*CC_B_*
0.321	0.169	0.373	0.334	0.171	0.192

**Table 2:** The ranges of the categories of proper waste management for the analyzed fractions of selectively collected waste.

Evaluation Category	*PC*	*G*	*P*	*WEEE*	*BW*	*B*
	Range					
I—the highest	<2.653–4.572>	<2.146–2.923>	<2.887–4.547>	<8.687–18.059>	<2.146–3.411>	<1.502–2.333>
II—high	<1759–2653)	<1.611–2.146)	<2.041–2.887)	<2.497–8.687)	<1.476–2.146)	<1.109–1.502)
III—average	<1.153–1.759)	<1.221–1.611)	<1289–2.041)	<1.390–2.497)	<1.030–1.476)	<0.771–1109)
IV—low	<0599–1153)	<0.839–1.221)	<0.562–1.289)	<0.629–1.390)	<0.657–1.030)	<0.421–0.771)
V—the lowest	<0.000–0.599)	<0.039–0.830)	<0.000–0.562)	<0.000–0.629)	<0.005–0.657)	<0.000–0.421)

**Table 3:** The number of counties in particular ranges in waste management correctness categories for the chosen fractions of the selected waste.

Evaluation Category	*PC*	*G*	*P*	*WEEE*	*BW*	*B*
I—the highest	15	17	30	2	10	33
II—high	39	62	65	34	58	77
III—average	80	107	88	65	95	100
IV—low	130	114	94	141	120	89
V—the lowest	116	80	103	138	97	81

## Supplementary Materials

The following are available online at https://www.mdpi.com/1660-4601/17/23/9107/s1, Figure S1: The administrative division of Poland, Table S1: The values of the location quotients (*LQ*) for selected waste in comparison with all municipal waste in counties in Poland, Table S2: The values of the location *quotients (LQ)* for the analyzed fractions of selected waste in comparison with all selected waste in counties in Poland.

## References

[1] Dyson B., Chang N.B. (2005). Forecasting municipal solid waste generation in a fast-growing urban region with system dynamics modeling. Waste Manag..

[2] Hryb W. (2015). Recykling odpadów komunalnych w Polsce-aktualny stan i perspektywy rozwoju (Recycling of municipal wastes in Poland-current status and development prospects). Arch. Gospod. Odpadami I Ochr. Środowiska.

[3] Green K.W., Zelbst P.J., Meacham J., Bhadauria V.S. (2012). Green supply chain management practices: Impact on performance. Supply Chain Manag.-Int. J..

[4] Kłos L. (2012). Gospodarka odpadami komunalnymi-wyzwanie XXI wieku (Municipal Waste Management-the Challenge of the XXI Century). Zesz. Nauk. Uniw. Szczecińskiego. Studia I Pr. Wydziału Nauk Ekon. I Zarządzania.

[5] Kaza S., Yao L., Bhada-Tata P., Van Woerden F. (2018). What a Waste 2.0: A Global Snapshot of Solid Waste Management to 2050.

[6] Wang X.M., Stanisavljevic N. (2019). Can waste management be hailed as a climate change mitigation leader?. Waste Manag. Res..

[7] Official Journal of the European Communities (2008). Directive 2008/98/EC of the European Parliament and of the Council of 19 November 2008 on Waste and Repealing Certain Directives. https://eur-lex.europa.eu/eli/dir/2008/98/oj.

[8] Official Journal of the European Communities (2018). Directive (EU) 2018/851 of the European Parliament and of the Council of 30 May 2018 Amending Directive 2008/98/EC on Waste. https://eur-lex.europa.eu/eli/dir/2018/851/oj.

[9] Central Statistical Office Bank Danych Lokalnych (Local Data Bank). https://bdl.stat.gov.pl/BDL/start.

[10] Internet System of Legal Acts Krajowy Plan Gospodarki Odpadami 2022 (National Waste Management Plan 2022). https://isap.sejm.gov.pl/isap.nsf/DocDetails.xsp?id=WMP20160000784.

[11] Internet System of Legal Acts Obwieszczenie Ministra Środowiska z Dnia 7 Października 2019 r. w Sprawie Ogłoszenia Jednolitego Tekstu Rozporządzenia Ministra Środowiska w Sprawie Szczegółowego Sposobu Selektywnego Zbierania Wybranych Frakcji Odpadów (The Decree of the Minister of the Environment Dated 7th October 2019 on Announcing the Consolidated Text of the Regulation of the Minister of the Environment on a Detailed way of Selective Collection of Given Waste Fractions). https://isap.sejm.gov.pl/isap.nsf/DocDetails.xsp?id=WDU20190002028.

[12] Internet System of Legal Acts Ustawa z Dnia 13 Września 1996 r. o Utrzymaniu Czystości i Porządku w Gminach (The Act of 13th September 1996 on the Cleanliness and Order in Counties). https://isap.sejm.gov.pl/isap.nsf/DocDetails.xsp?id=WDU20200001439.

[13] Internet System of Legal Acts Ustawa z Dnia 14 Grudnia 2012 r. o Odpadach (The Act of 14th December 2012 on the Waste). https://isap.sejm.gov.pl/isap.nsf/DocDetails.xsp?id=WDU20200000797.

[14] Internet System of Legal Acts Ustawa z Dnia 11 Września 2015 r. o Zużytym Sprzęcie Elektrycznym i Elektronicznym (The Act of 11th September 2015 on Waste Electrical and Electronic Waste). https://isap.sejm.gov.pl/isap.nsf/DocDetails.xsp?id=WDU20200001893.

[15] Internet System of Legal Acts RozporząDzenie Ministra Klimatu z Dnia 2 Stycznia 2020 r. w Sprawie Katalogu Odpadów (The Decree of the Minister of Climate of the 2nd January 2020 on Waste Catalogue). https://isap.sejm.gov.pl/isap.nsf/DocDetails.xsp?id=WDU20200000010.

[16] Alankiewicz T.P. (2009). Skuteczność Funkcjonowania Gospodarki Odpadami na Przykładzie Jednostek Samorządowych Województwa Wielkopolskiego. Praca Doktorska (Ph.D. Thesis).

[17] Deluga W. (2018). Gospodarka odpadami w świadomości społeczeństwa (Waste Management in Public Awarenes). Rocz. Ochr. Środowiska.

[18] Wengierek M. (2015). Analiza i ocena gospodarki odpadami komunalnymi w wybranych miastach regionu śląskiego (Analysis and Evaluation of Municipal Waste Management in Selected Cities of Silesia Region). Zesz. Nauk. Politech. Śląskiej.

[19] Pawlewicz A., Gotkiewicz W., Mickiewicz B. (2019). Awareness of Waste Management in Single-Family and Multi-Family Housing Estates on the Example of Olsztyn. Rocz. Ochr. Środowiska.

[20] Senetra A., Krzywnicka I., Tuyet M.D.T. (2019). The Analysis and the Evaluation of Municipal Waste Management in Voivodship Cities in Poland. Rocz. Ochr. Srodowiska.

[21] Lewandowska A., Szymańska D. (2019). Municipal waste recycling in big cities in Poland in the context of ecologisation. Bull. Geogr. Socio Econ. Ser..

[22] Tausova M., Mihalikova E., Culkova K., Stehlikova B., Taus P., Kudelas D., Strba L., Domaracka L. (2020). Analysis of Municipal Waste Development and Management in Self-Governing Regions of Slovakia. Sustainability.

[23] Castillo-Gimenez J., Montanes A., Picazo-Tadeo A.J. (2019). Performance and convergence in municipal waste treatment in the European Union. Waste Manag..

[24] Ministry of the Environment (2018). Trackingowe Badanie Świadomości i Zachowań Ekologicznych Mieszkańców Polski (Tracking Analysis of the Awareness and Ecological Behavior of the Inhabitants in Poland).

[25] De Feo G., Ferrara C., Iannone V., Parente P. (2019). Improving the efficacy of municipal solid waste collection with a communicative approach based on easily understandable indicators. Sci. Total Environ..

[26] Lorek A. (2015). Ocena systemu gospodarki odpadami komunalnymi województwa śląskiego w opinii konsumentów (Assessment of household waste management system in silesian voivodeship in consumer opinion). Studia Ekon..

[27] Cole C., Gnanapragasam A., Cooper T., Singh J. (2019). An assessment of achievements of the WEEE Directive in promoting movement up the waste hierarchy: Experiences in the UK. Waste Manag..

[28] Domański R. (1998). Zasady Geografii Społeczno-Ekonomicznej.

[29] Zioło Z. (1968). Wskaźnik koncentracji jako miernik zróżnicowania przestrzennego-na przykładzie rozmieszczenia ludności województwa rzeszowskiego (The concentration coefficient as the measurement of spatial differentiation–on the example of the population distribution of Rzeszów Voivodeship). Rocz. Nauk.-Dydakt. Wsp W Krakowie.

[30] Kostrubiec B., Chojnicki Z. (1977). Metody badania koncentracji przestrzennej. Metody Ilościowe I Modele W Geografii.

[31] Senetra A., Cieślak I. (2004). Kartograficzne Aspekty Oceny I Waloryzacji Przestrzeni.

[32] Jenks G.F. (1967). The Data Model Concept in Statistical Mapping. Int. Yearb. Cartogr..

[33] Rey S.J., Stephens P., Laura J. (2017). An evaluation of sampling and full enumeration strategies for Fisher Jenks classification in big data settings. Trans. Gis.

[34] Shen S.T., Zhang S.J., Fan M., Wang Q. (2017). Classification of plant functional types based on the nutrition traits: A case study on alpine meadow community in the Zoige Plateau. J. Mt. Sci..

[35] Yalcin M., Gul F.K. (2017). A GIS-based multi criteria decision analysis approach for exploring geothermal resources: Akarcay basin (Afyonkarahisar). Geothermics.

