# Supplementary material for: The Evaluation of Municipal Waste in Counties in Poland with the Use of the Theory of Phenomena Spatial Concentration
Source: Int J Environ Res Public Health. 2020 Dec 6;17(23):9107. doi: 10.3390/ijerph17239107 (PMC7731399; doi:10.3390/ijerph17239107)
Supplement: Supplementary file 1 [file ijerph-17-09107-s001.zip › Supplementary materials/Figure.S1.pdf]

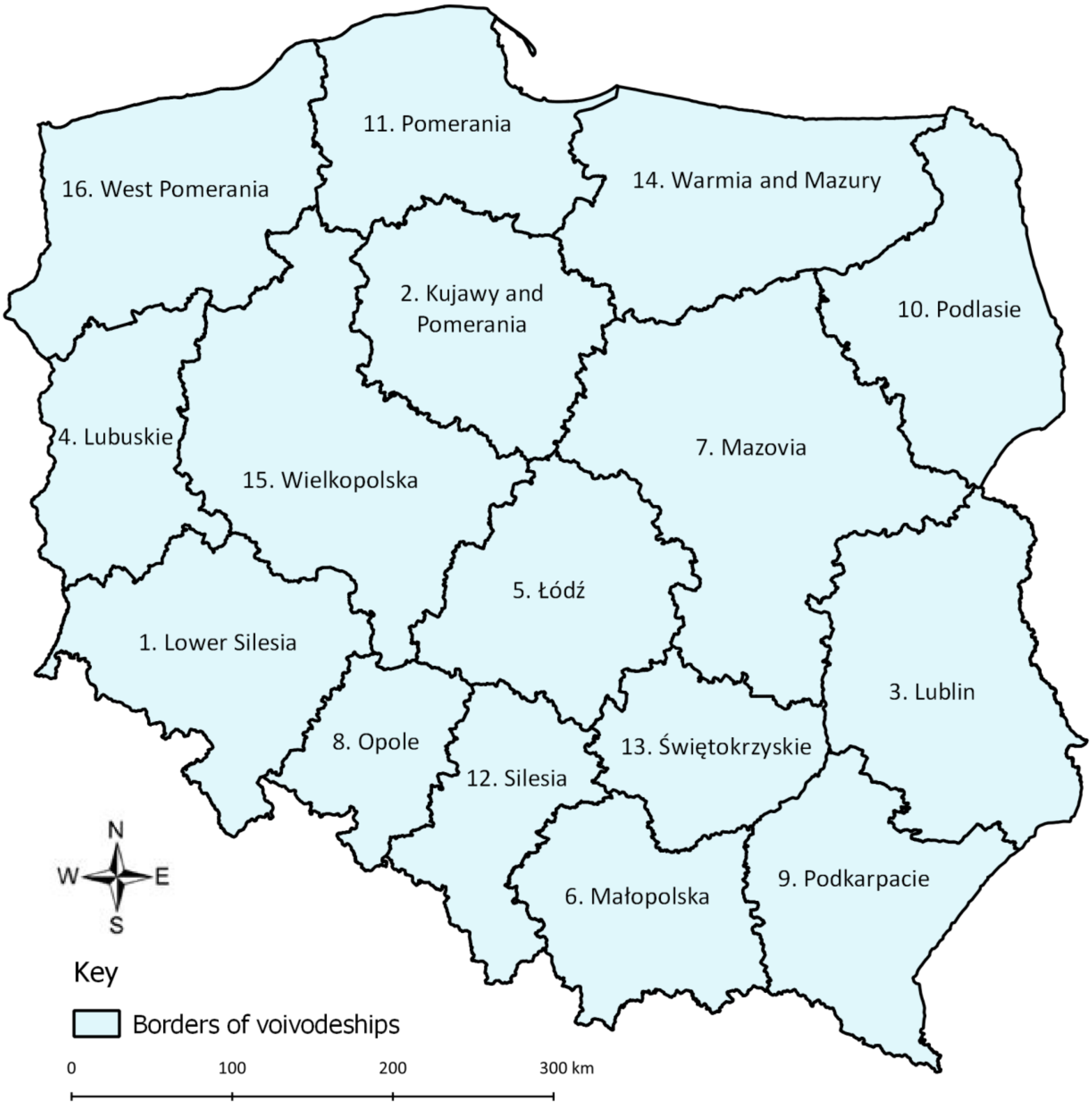

Fig. 1. The administrative division of Poland – voivodeships

Source: Own elaboration

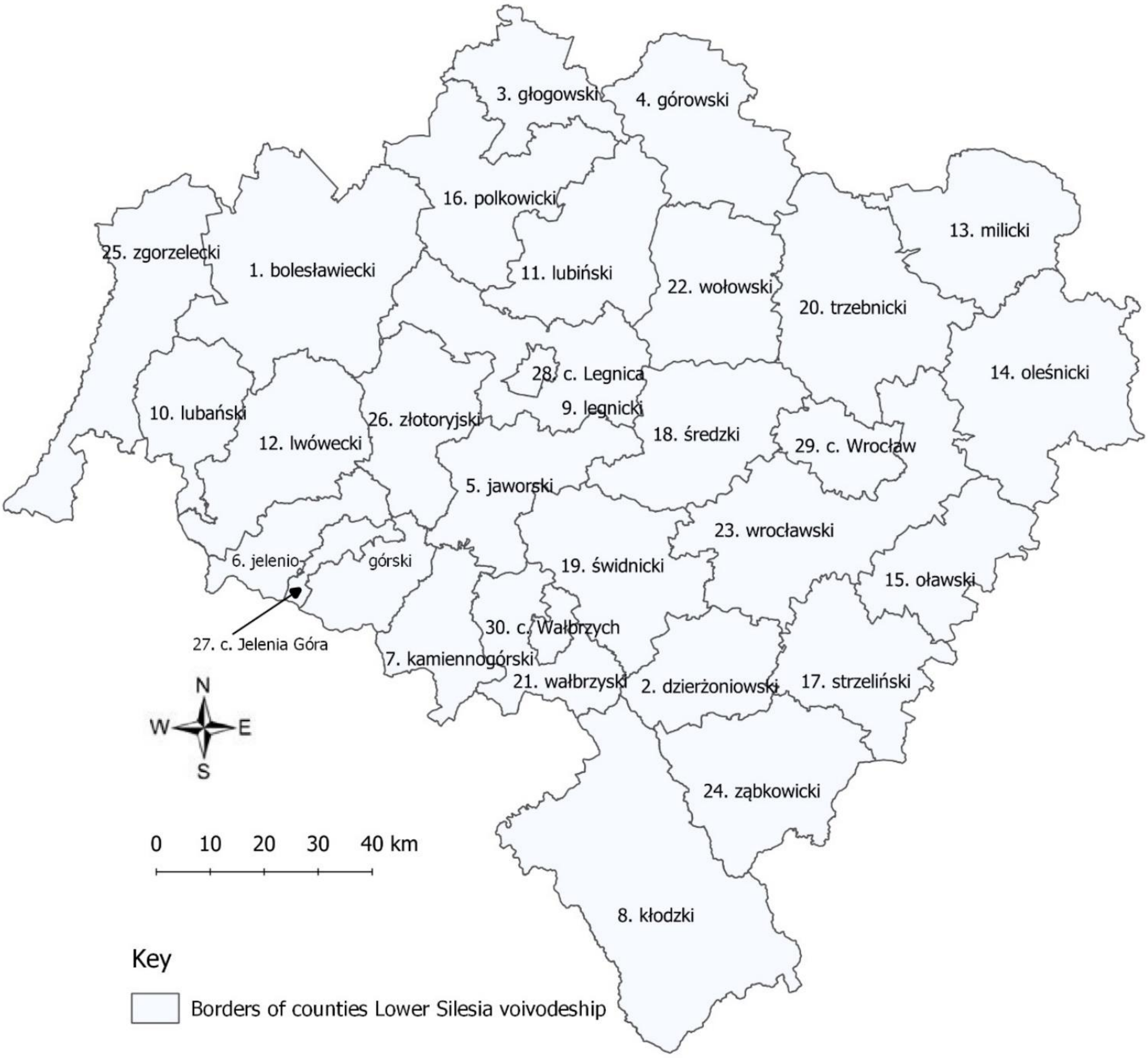

Fig. 2. Counties in Lower Silesia voivodeship

Source: Own elaboration

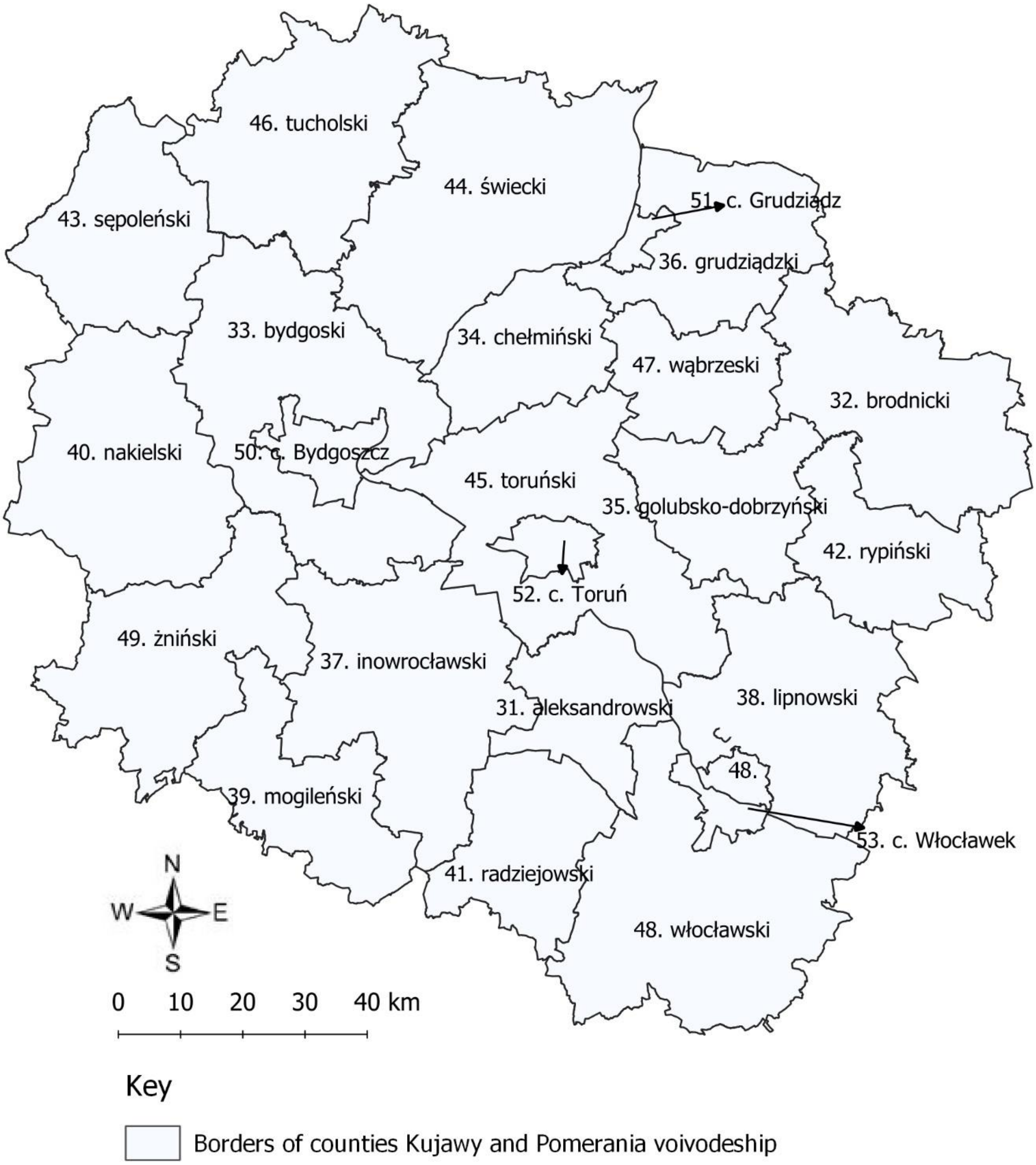

Fig .3. Counties in Kujawy and Pomerania voivodeship

Source: Own elaboration

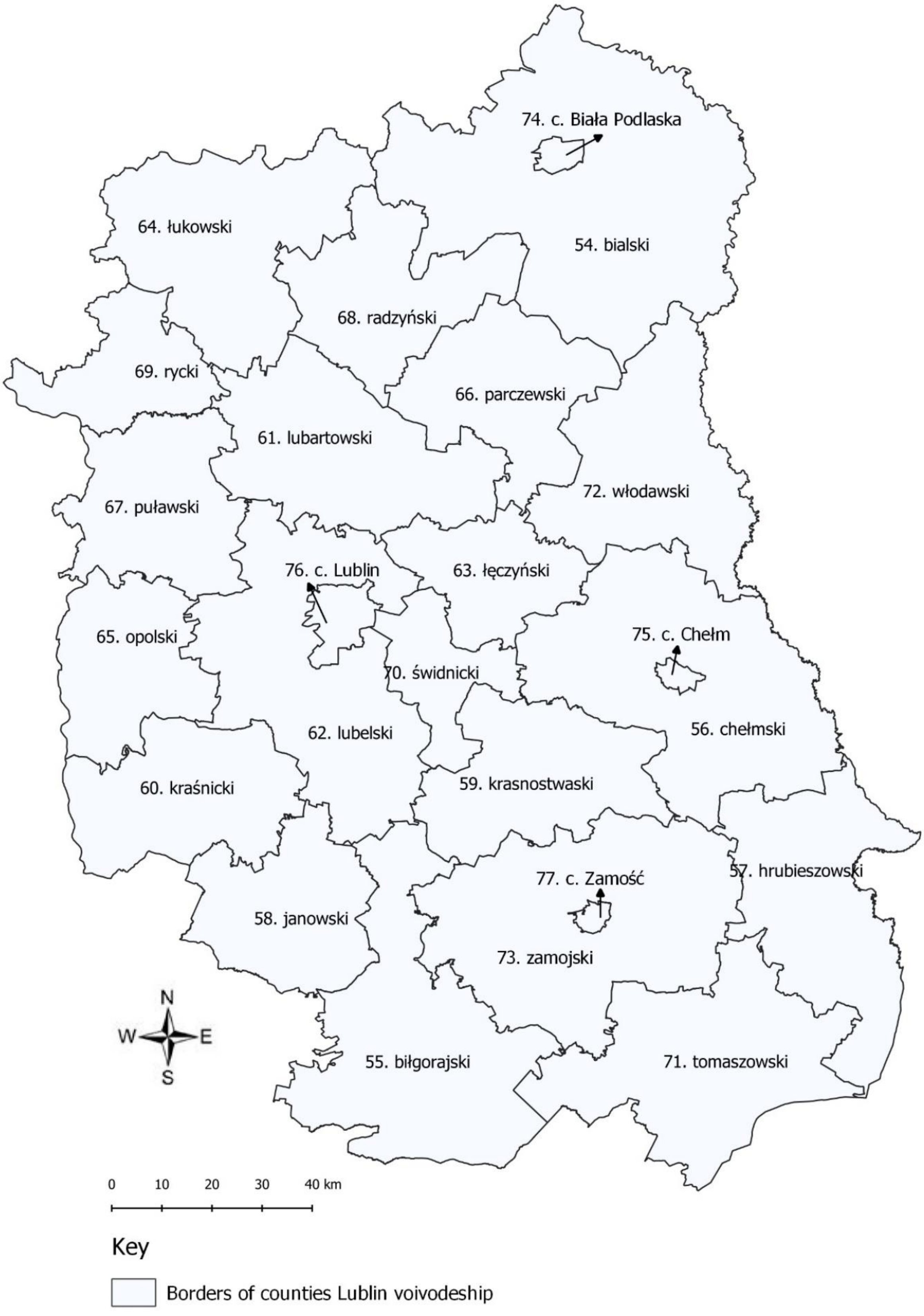

Fig.4. Counties in Lublin voivodeship

Source: Own elaboration

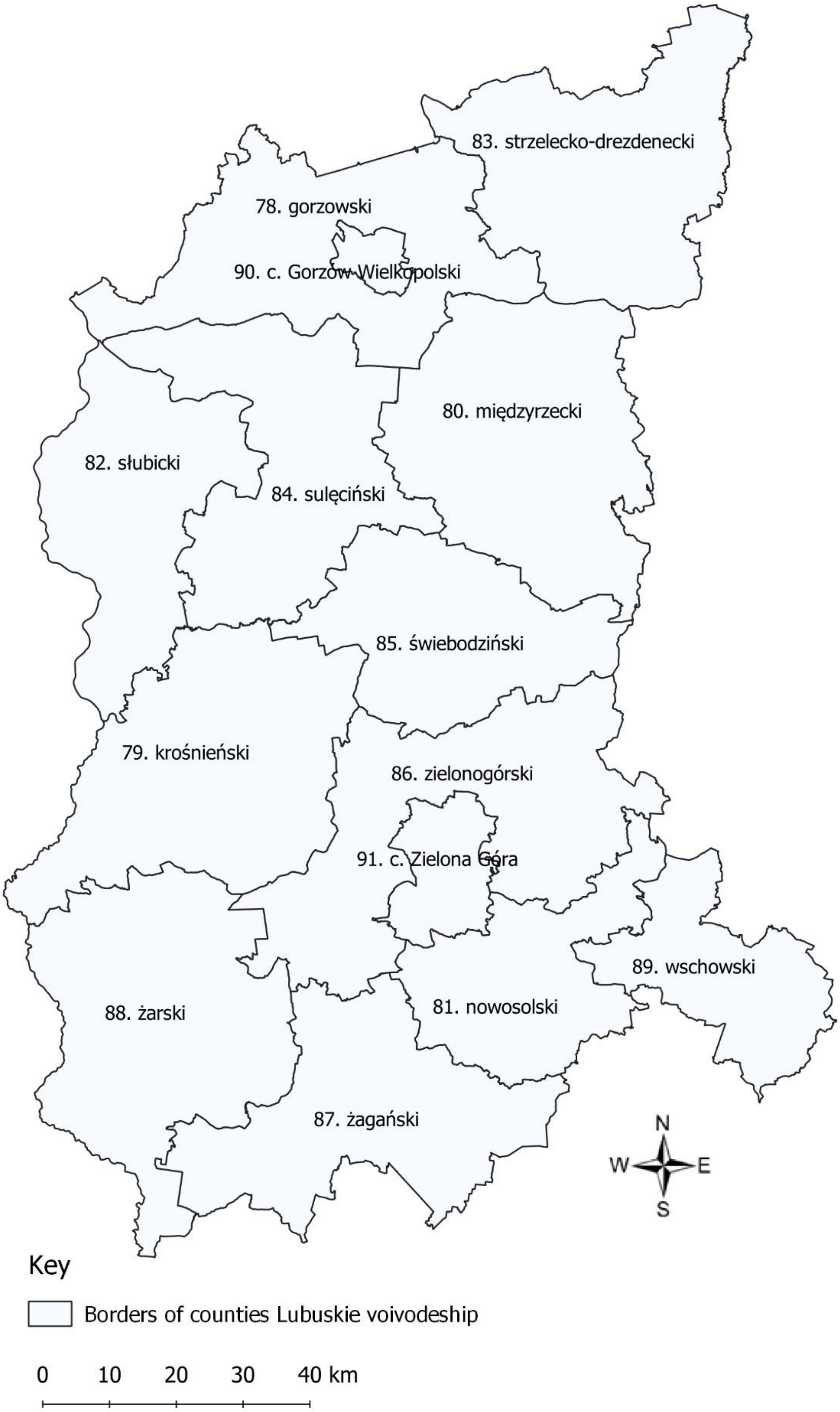

Fig. 5. Counties in Lubuskie voivodeship

Source: Own elaboration

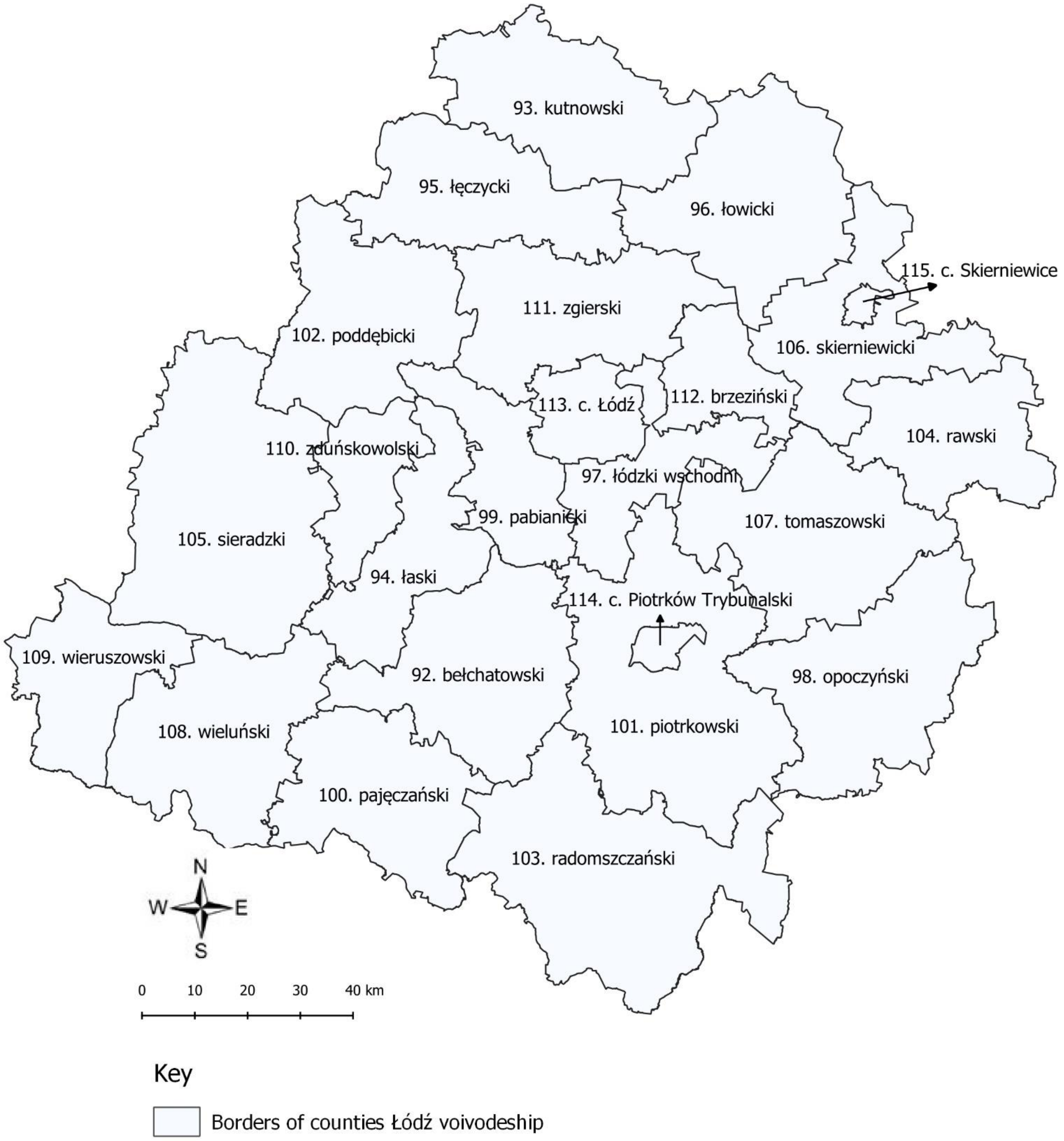

Fig. 6. Counties in Łódź voivodeship

Source: Own elaboration

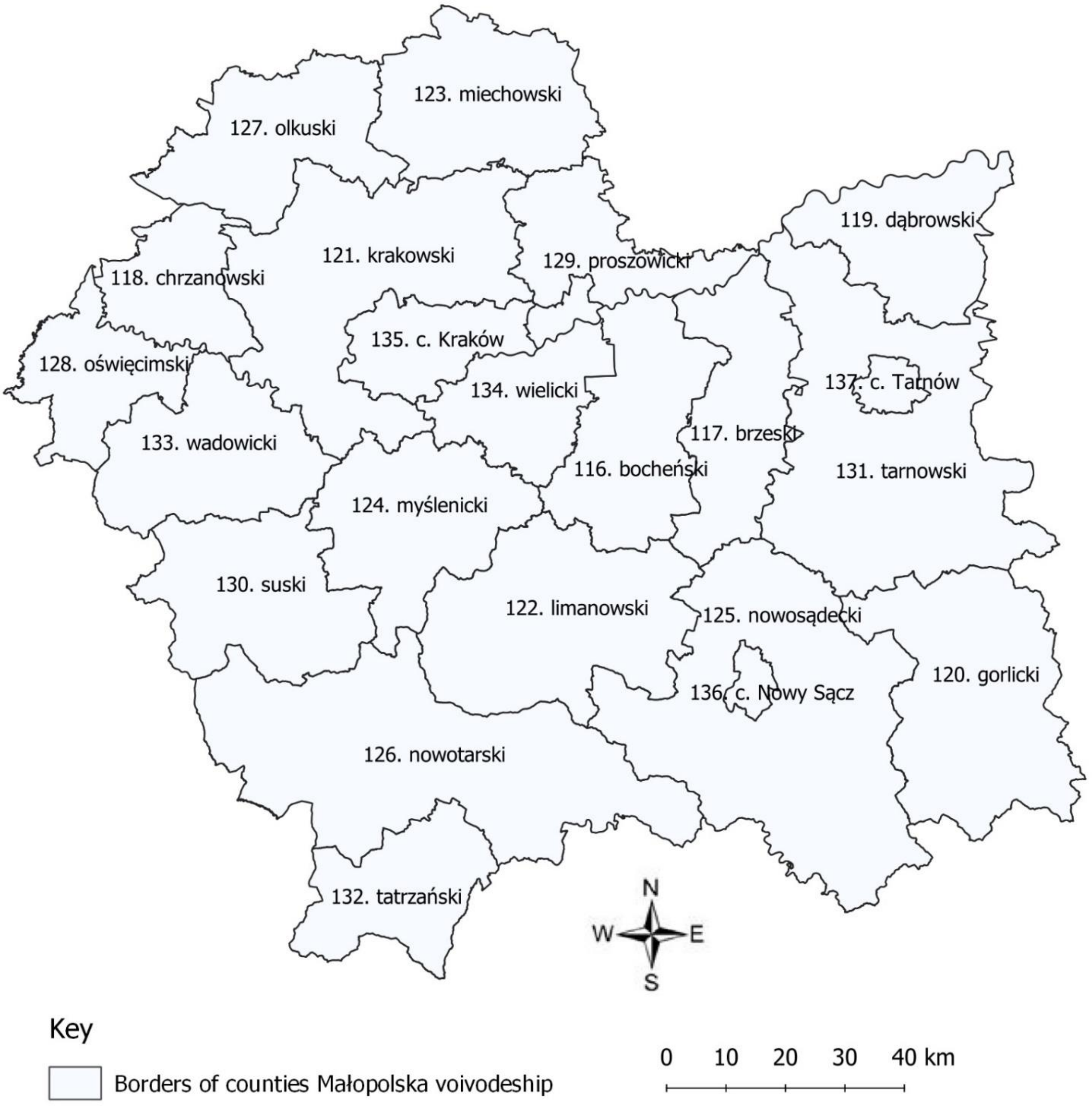

Fig. 7. Counties in Małopolska voivodeship

Source: Own elaboration

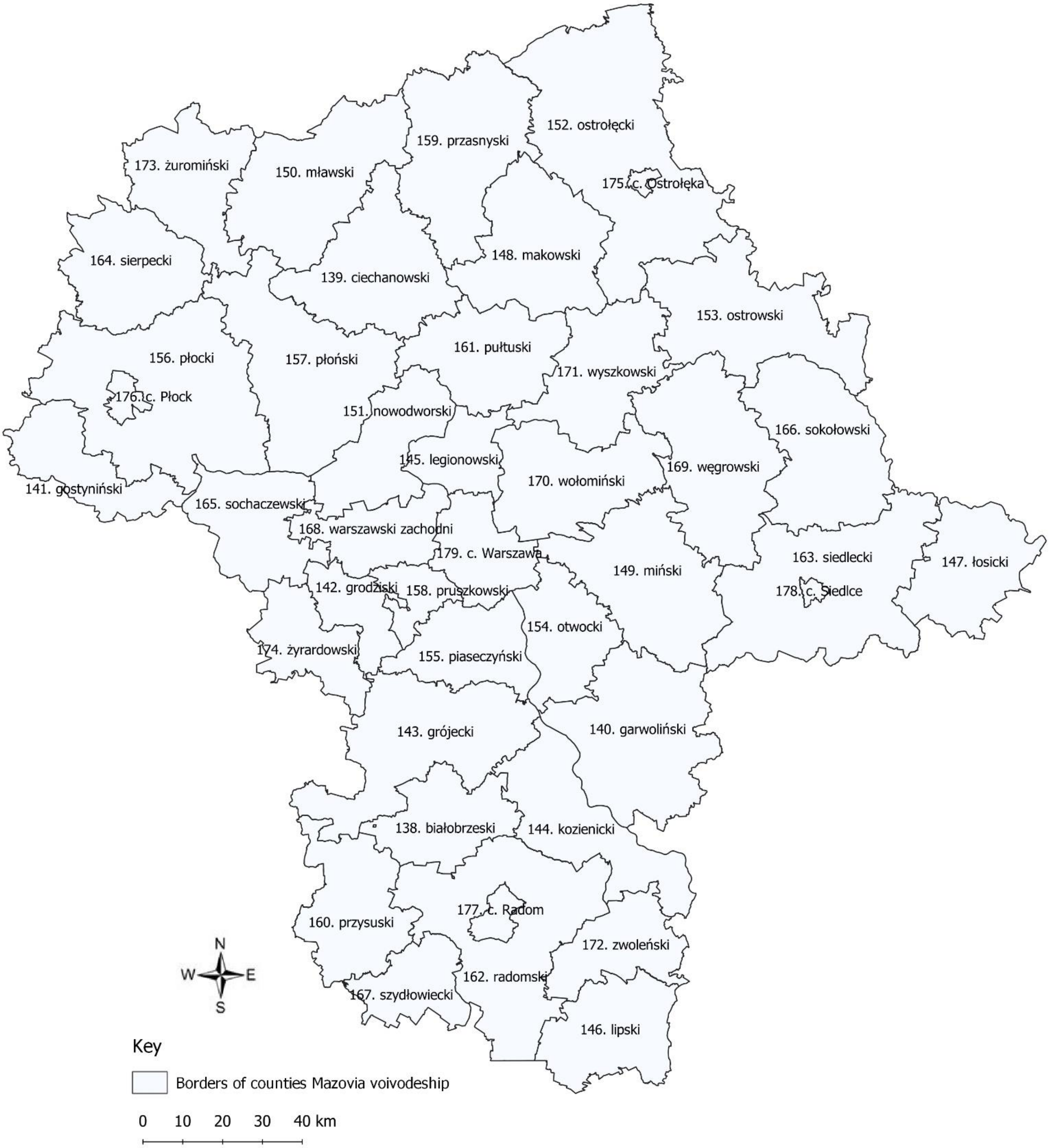

Fig. 8. Counties in Mazovia Voivodeship

Source: Own elaboration

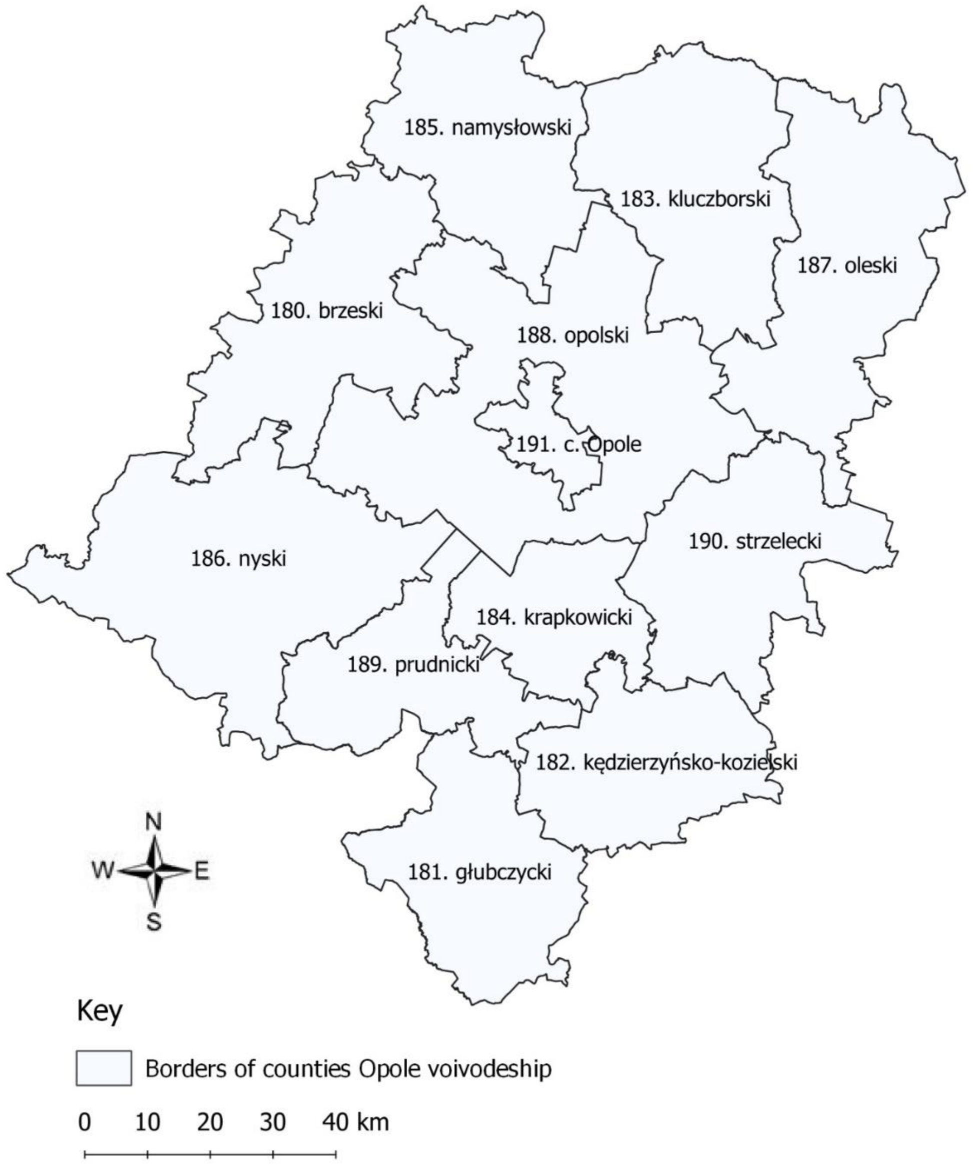

Fig. 9. Counties in Opole voivodeship

Source: Own elaboration

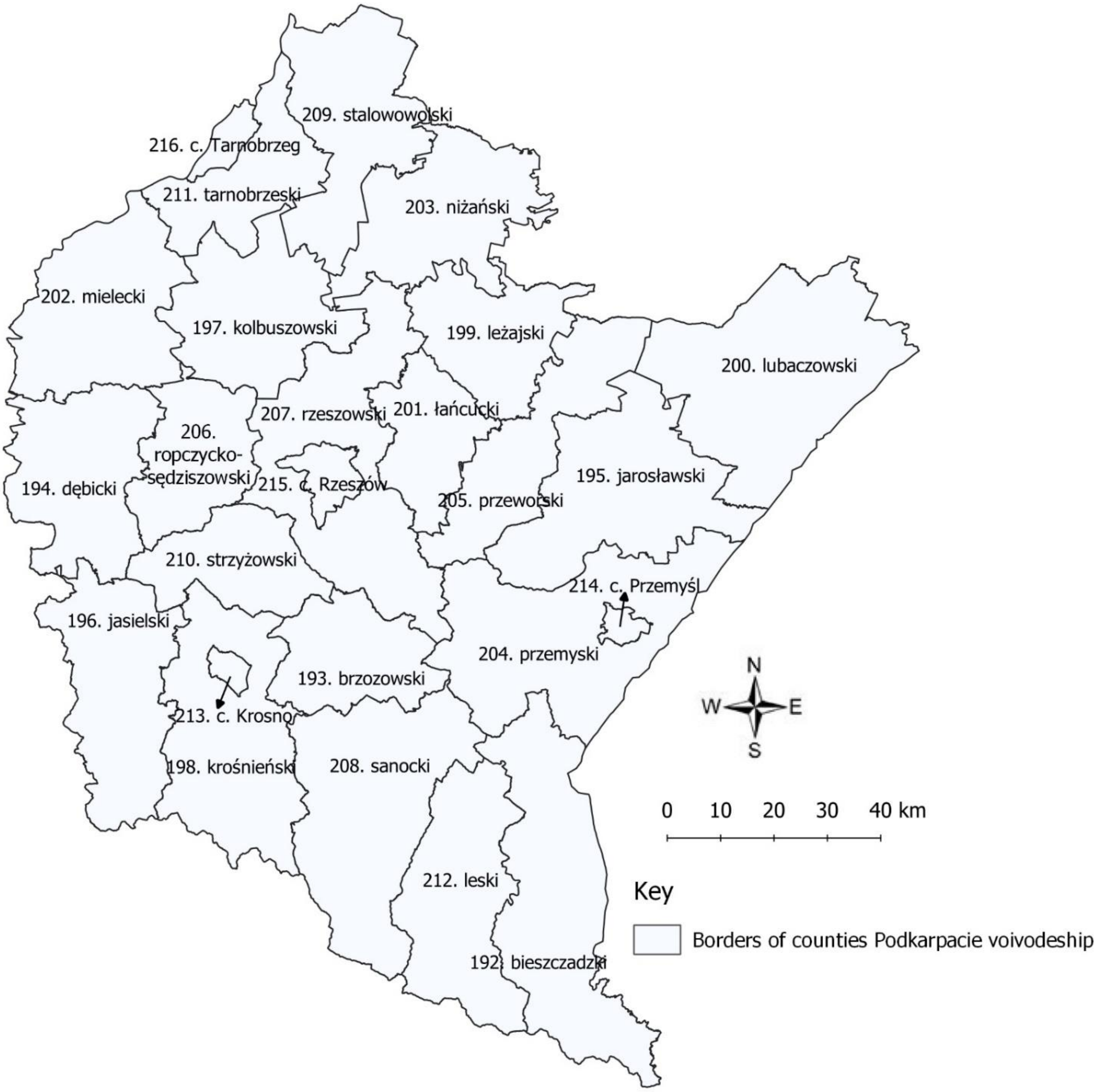

Fig. 10. Counties in Podkarpacie voivodeship

Source: Own elaboration

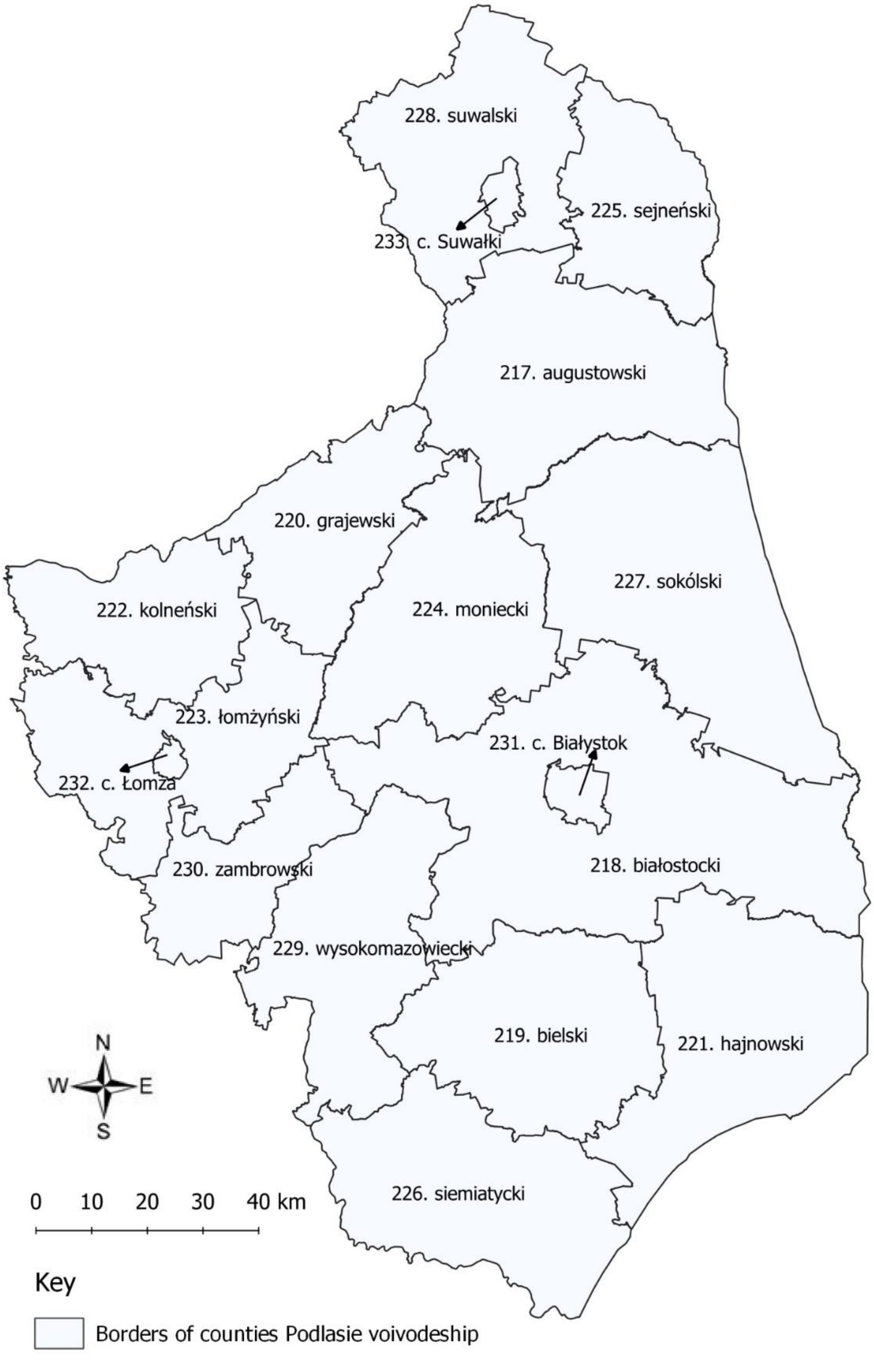

Fig. 11. Counties in Podlasie voivodeship

Source: Own elaboration

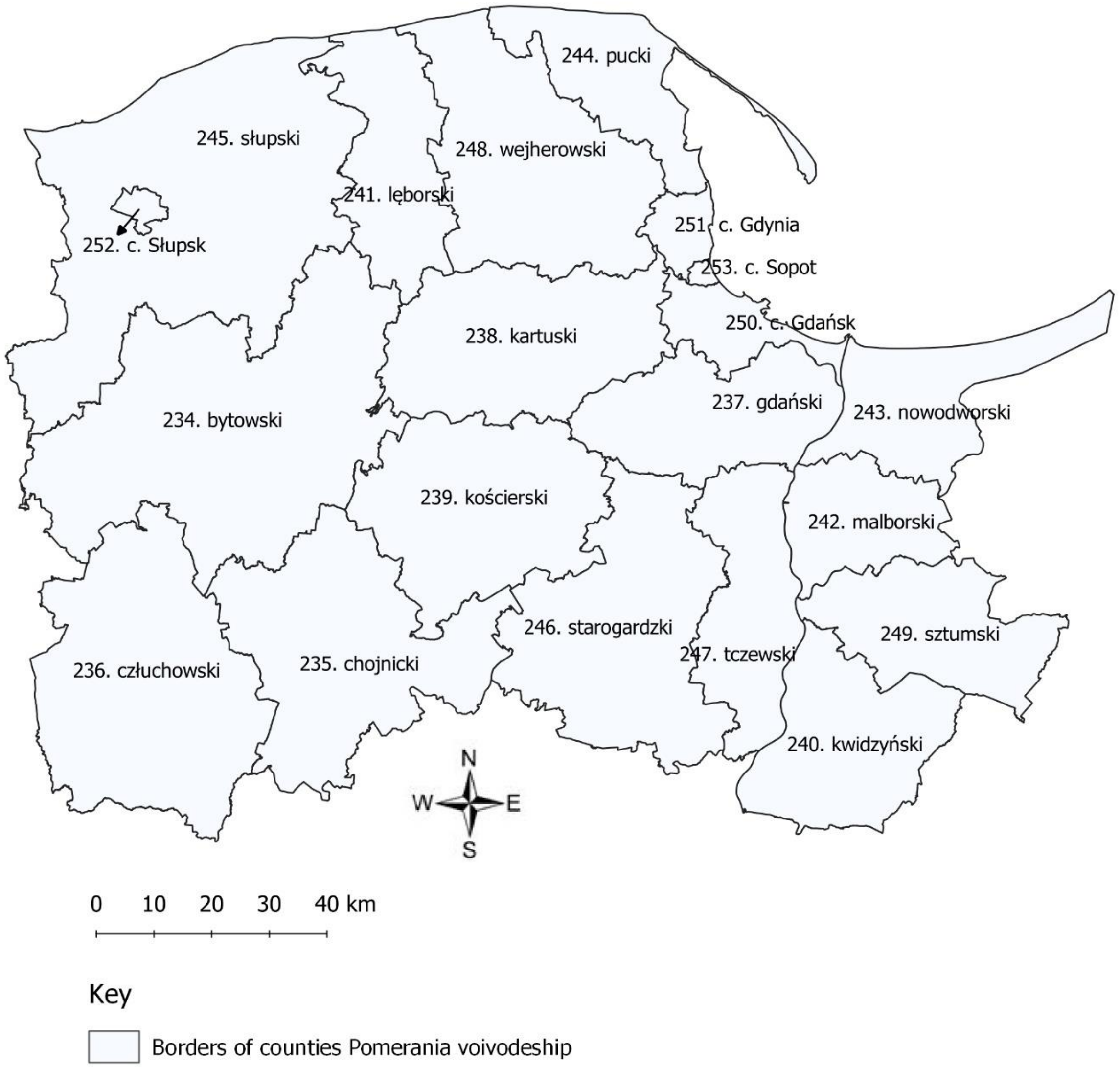

Fig. 12. Counties in Pomerania voivodeship

Source: Own elaboration

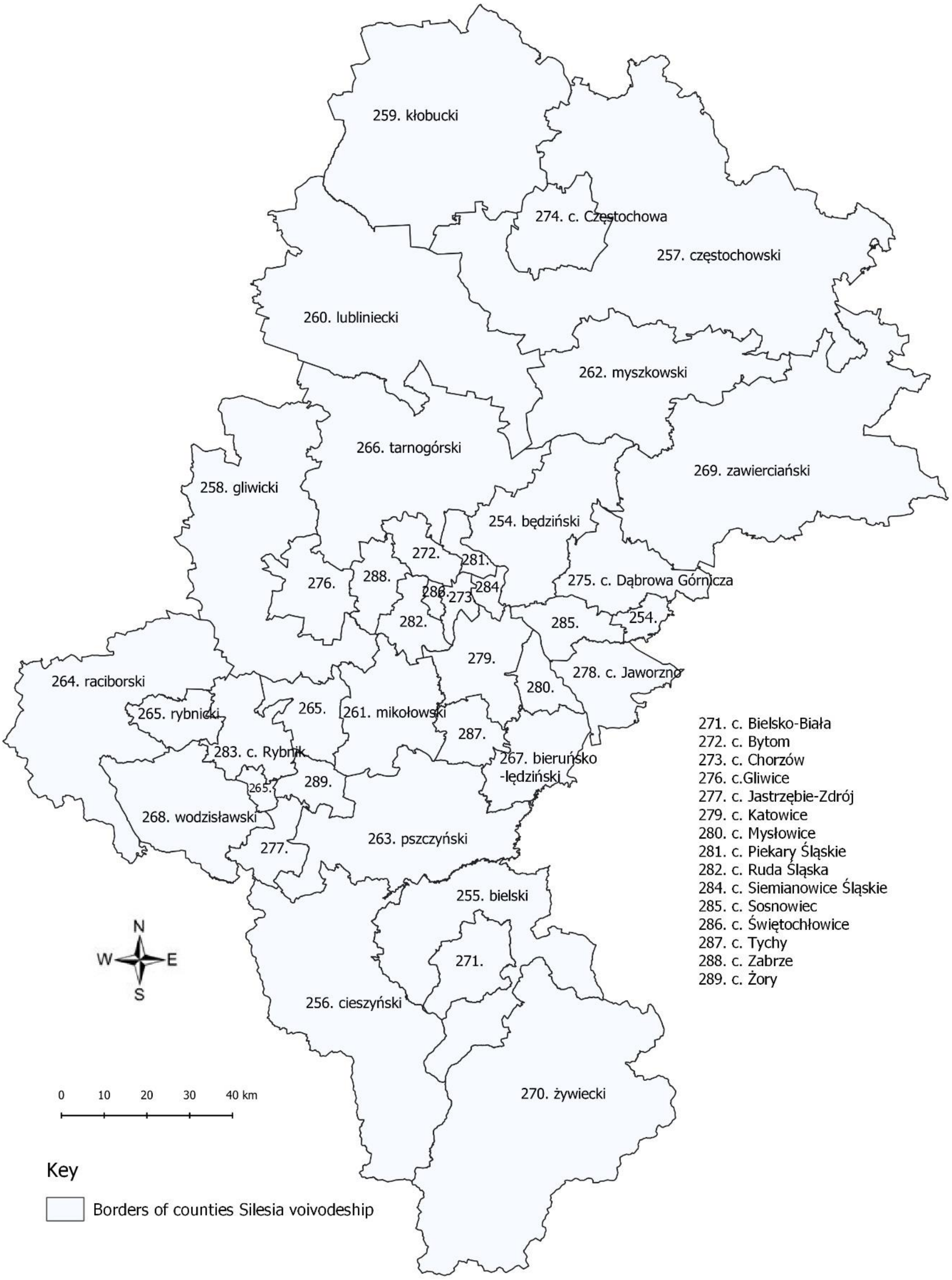

Fig. 13. Counties in Silesia voivodeship

Source: Own elaboration

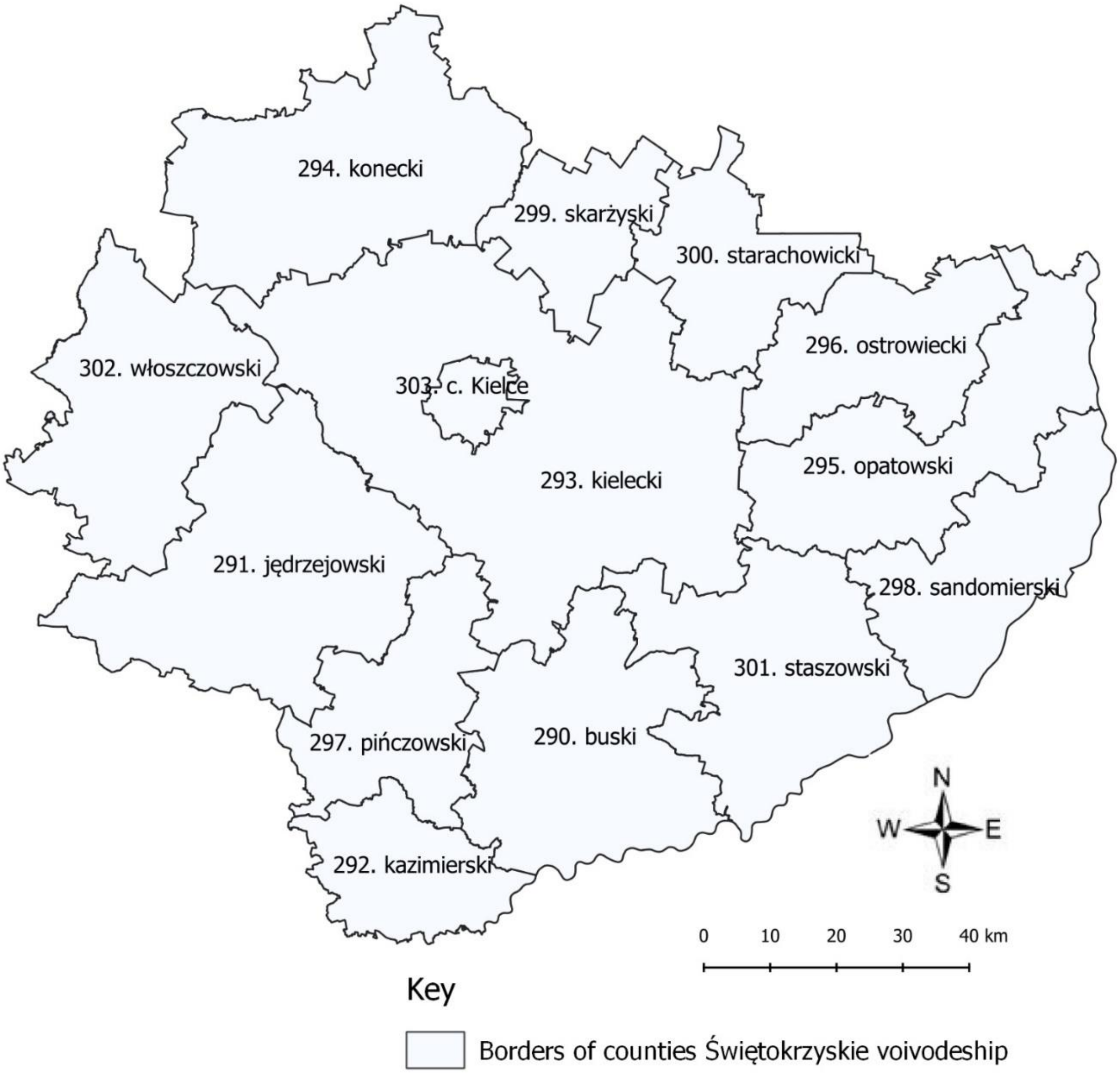

Fig. 14. Counties in Świętokrzyskie voivodeship

Source: Own elaboration

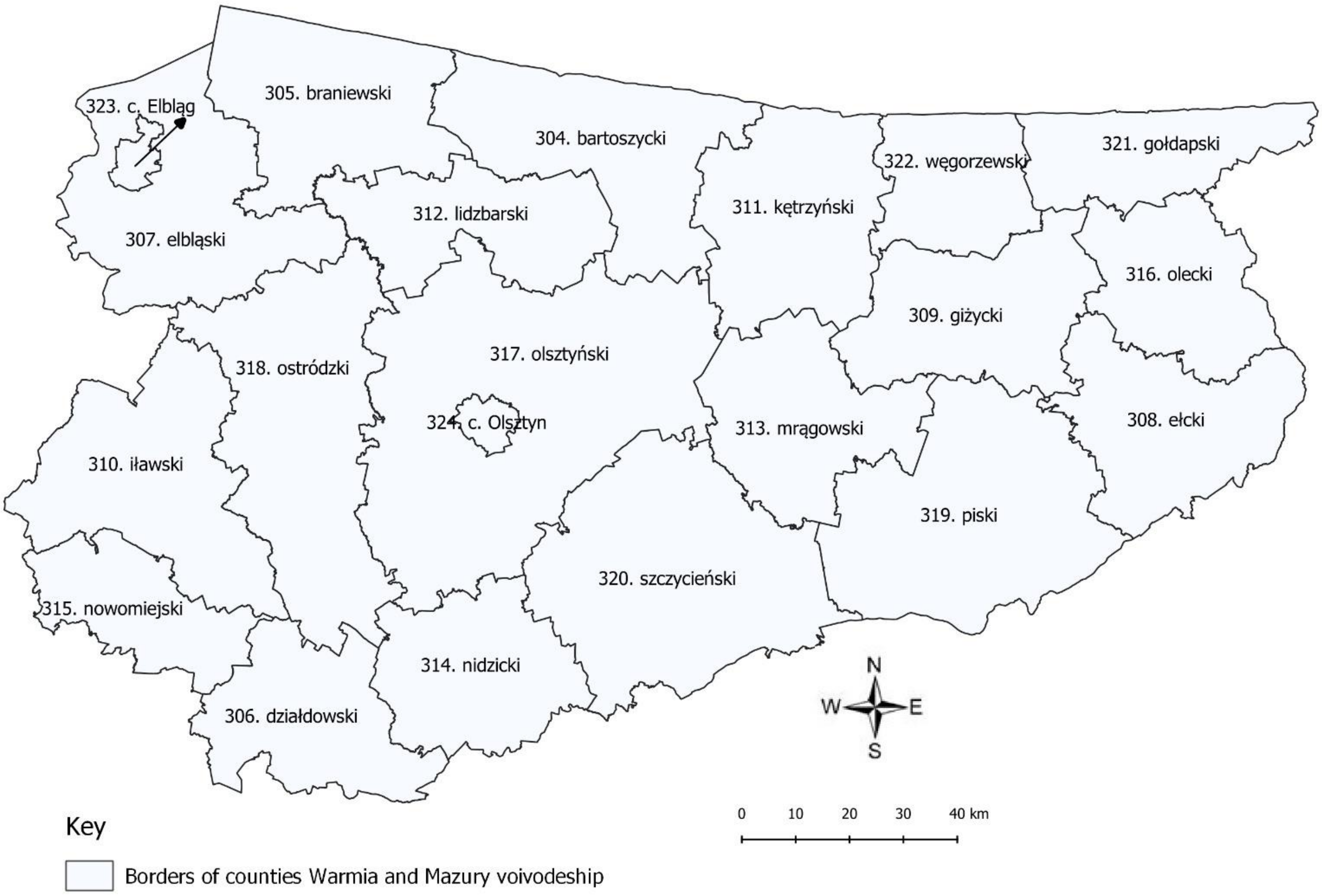

Fig. 15. Counties in Warmia and Mazury voivodeship

Source: Own elaboration

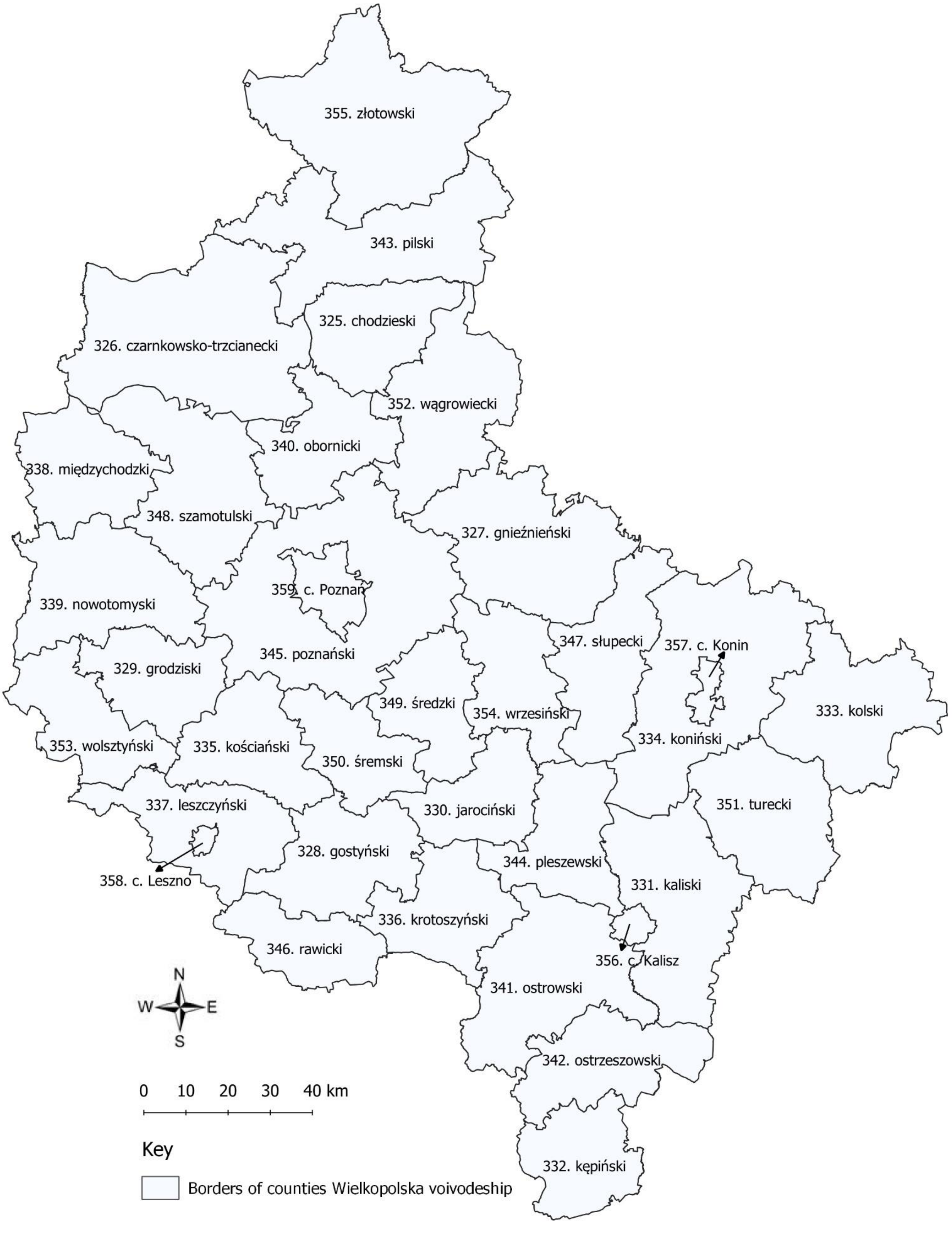

Fig. 16. Counties in Wielkopolska voivodeship

Source: Own elaboration

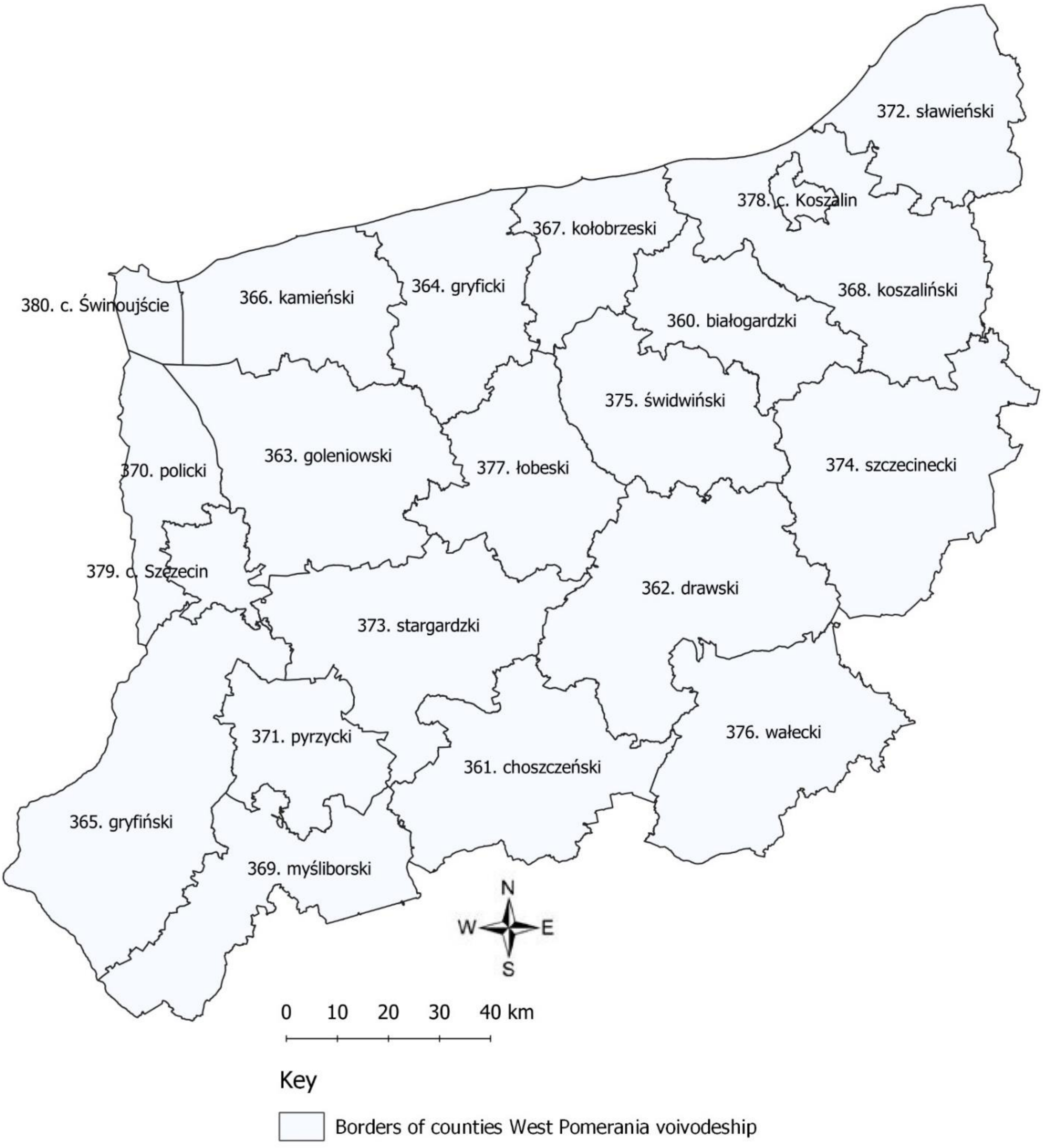

Fig. 17. Counties in West Pomerania voivodeship

Source: Own elaboration
