# Supplementary material for: The Evaluation of Municipal Waste in Counties in Poland with the Use of the Theory of Phenomena Spatial Concentration
Source: Int J Environ Res Public Health. 2020 Dec 6;17(23):9107. doi: 10.3390/ijerph17239107 (PMC7731399; doi:10.3390/ijerph17239107)
Supplement: Supplementary file 1 [file ijerph-17-09107-s001.zip › Supplementary materials/Tabele.S1.pdf]

Table S1. The values of the location quotients (*LQ*) for selected waste in comparison with all municipal waste in counties in Poland

No. voivodeship	Voivodeship	Counties	Location quotient (*LQ*)
1.	Lower Silesia	1. bolesławiecki	1.012
		2. dzierżoniowski	0.545
		3. głogowski	0.631
		4. górowski	0.625
		5. jaworski	0.630
		6. jeleniogórski	0.894
		7. kamiennogórski	0.650
		8. kłodzki	0.592
		9. legnicki	0.978
		10. lubański	0.938
		11. lubiński	0.666
		12. lwówecki	1.061
		13. milicki	0.897
		14. oleśnicki	1.109
		15. oławski	0.393
		16. polkowicki	0.911
		17. strzeliński	0.832
		18. średzki	0.785
		19. świdnicki	0.772
		20. trzebnicki	1.223
		21. wałbrzyski	0.590
		22. wołowski	1.015
		23. wrocławski	1.107
		24. ząbkowicki	0.590
		25. zgorzelecki	0.669
		26. złotoryjski	0.732
		27. c. Jelenia Góra	0.488
		28. c. Legnica	0.836

		29. c. Wrocław	1.049
		30. c. Wałbrzych	0.073
2.	Kujawy and Pomerania	31. aleksandrowski	1.060
		32. brodnicki	0.855
		33. bydgoski	1.183
		34. chełmiński	0.727
		35. golubsko-dobrzyński	0.756
		36. grudziądzki	0.766
		37. inowrocławski	0.816
		38. lipnowski	1.406
		39. mogileński	0.877
		40. nakielski	0.675
		41. radziejowski	0.864
		42. rypiński	0.752
		43. sępoleński	1.018
		44. świecki	0.867
		45. toruński	1.106
		46. tucholski	1.363
		47. wąbrzeski	1.041
		48. włocławski	1.511
		49. żniński	0.746
		50. c. Bydgoszcz	1.235
		51. c. Grudziądz	1.064
		52. c. Toruń	0.705
		53. c. Włocławek	0.931
3.	Lublin	54. bialski	1.821
		55. biłgorajski	1.204
		56. chełmski	1.719
		57. hrubieszowski	0.852
		58. janowski	0.816

		59. krasnostawski	0.872
		60. kraśnicki	1.057
		61. lubartowski	1.036
		62. lubelski	1.070
		63. łęczyński	0.996
		64. łukowski	1.850
		65. opolski	0.964
		66. parczewski	1.896
		67. puławski	1.101
		68. radzyński	1.956
		69. rycki	0.960
		70. świdnicki	0.996
		71. tomaszowski	1.302
		72. włodawski	1.211
		73. zamojski	1.212
		74. c. Biała Podlaska	0.751
		75. c. Chełm	1.218
		76. c. Lublin	0.971
		77. c. Zamość	0.987
4.	Lubuskie	78. gorzowski	0.783
		79. krośnieński	0.768
		80. międzyrzecki	0.614
		81. nowosolski	1.338
		82. słubicki	0.712
		83. strzelecko-drezdenecki	0.424
		84. sulęciński	1.019
		85. świebodziński	0.666
		86. zielonogórski	0.990
		87. żagański	0.633
		88. żarski	0.832

		89. wschowski	1.126
		90. c. Gorzów Wielkopolski	0.729
		91. c. Zielona Góra	0.727
5.	Łódź	92. bełchatowski	1.279
		93. kutnowski	1.158
		94. łaski	1.530
		95. łęczycki	0.638
		96. łowicki	0.824
		97. łódzki wschodni	1.144
		98. opoczyński	0.917
		99. pabianicki	1.424
		100. pajęczański	1.016
		101. piotrkowski	1.212
		102. poddębicki	0.543
		103. radomszczański	0.845
		104. rawski	0.653
		105. sieradzki	1.000
		106. skierniewicki	1.483
		107. tomaszowski	0.727
		108. wieluński	0.979
		109. wieruszowski	1.156
		110. zduńskowolski	1.028
		111. zgierski	1.432
		112. brzeziński	1.229
		113. c. Łódź	1.025
		114. c. Piotrków Trybunalski	0.949
		115. c. Skierniewice	0.888
6.	Małopolska	116. bocheński	1.268
		117. brzeski	1.065

		118. chrzanowski	0.829
		119. dąbrowski	0.744
		120. gorlicki	0.763
		121. krakowski	1.124
		122. limanowski	1.020
		123. miechowski	1.167
		124. myślenicki	1.869
		125. nowosądecki	1.146
		126. nowotarski	1.005
		127. olkuski	0.856
		128. oświęcimski	0.809
		129. proszowicki	0.932
		130. suski	1.230
		131. tarnowski	1.102
		132. tatrzański	0.867
		133. wadowicki	1.032
		134. wielicki	1.075
		135. c. Kraków	1.286
		136. c. Nowy Sącz	0.745
		137. c. Tarnów	1.132
7.	Mazovia	138. białobrzeski	0.971
		139. ciechanowski	0.665
		140. garwoliński	1.519
		141. gostyniński	1.012
		142. grodziski	1.227
		143. grójecki	0.748
		144. kozienicki	1.072
		145. legionowski	1.312
		146. lipski	1.192
		147. łosicki	1.119

		148. makowski	0.984
		149. miński	1.156
		150. mławski	0.537
		151. nowodworski	1.002
		152. ostrołęcki	0.617
		153. ostrowski	0.796
		154. otwocki	1.212
		155. piaseczyński	1.155
		156. płocki	0.984
		157. płoński	0.540
		158. pruszkowski	1.010
		159. przasnyski	0.732
		160. przysuski	0.814
		161. pułtuski	0.731
		162. radomski	1.236
		163. siedlecki	1.541
		164. sierpecki	0.543
		165. sochaczewski	1.330
		166. sokołowski	1.288
		167. szydłowiecki	0.731
		168. warszawski zachodni	1.186
		169. węgrowski	1.304
		170. wołomiński	1.184
		171. wyszkowski	1.465
		172. zwoleński	1.349
		173. żuromiński	0.370
		174. żyrardowski	0.718
		175. c. Ostrołęka	0.836
		176. c. Płock	0.692
		177. c. Radom	0.971

		178. c. Siedlce	0.864
		179. c. Warszawa	0.767
8.	Opole	180. brzeski	1.017
		181. głubczycki	1.299
		182. kędzierzyńsko-kozielski	1.183
		183. kluczborski	0.951
		184. krapkowicki	1.507
		185. namysłowski	0.668
		186. nyski	0.746
		187. oleski	1.147
		188. opolski	1.255
		189. prudnicki	1.050
		190. strzelecki	1.346
		191. c. Opole	1.248
9.	Podkarpacie	192. bieszczadzki	0.861
		193. brzozowski	1.462
		194. dębicki	1.066
		195. jarosławski	0.734
		196. jasielski	0.981
		197. kolbuszowski	0.930
		198. krośnieński	1.364
		199. leżajski	1.189
		200. lubaczowski	1.159
		201. łańcucki	0.931
		202. mielecki	0.588
		203. niżański	1.308
		204. przemyski	0.518
		205. przeworski	0.805
		206. ropczycko-sędziszowski	0.773

		207. rzeszowski	1.093
		208. sanocki	1.017
		209. stalowowolski	0.947
		210. strzyżowski	1.621
		211. tarnobrzeski	0.623
		212. leski	0.781
		213. c. Krosno	0.979
		214. c. Przemyśl	0.296
		215. c. Rzeszów	1.133
		216. c. Tarnobrzeg	0.507
10.	Podlasie	217. augustowski	0.674
		218. białostocki	0.889
		219. bielski	1.207
		220. grajewski	0.603
		221. hajnowski	1.107
		222. kolneński	0.488
		223. łomżyński	0.614
		224. moniecki	0.933
		225. sejneński	0.926
		226. siemiatycki	1.037
		227. sokólski	0.626
		228. suwalski	0.598
		229. wysokomazowiecki	0.632
		230. zambrowski	0.700
		231. c. Białystok	1.412
		232. c. Łomża	0.526
		233. c. Suwałki	0.458
11.	Pomerania	234. bytowski	0.946
		235. chojnicki	0.927

		236. człuchowski	1.021
		237. gdański	1.056
		238. kartuski	0.859
		239. kościerski	1.206
		240. kwidzyński	1.164
		241. lęborski	0.987
		242. malborski	0.761
		243. nowodworski	0.673
		244. pucki	1.373
		245. słupski	1.075
		246. starogardzki	1.119
		247. tczewski	0.656
		248. wejherowski	1.052
		249. sztumski	0.771
		250. c. Gdańsk	1.113
		251. c. Gdynia	0.885
		252. c. Słupsk	0.613
		253. c. Sopot	1.018
12.	Silesia	254. będziński	0.935
		255. bielski	2.161
		256. cieszyński	1.771
		257. częstochowski	1.334
		258. gliwicki	1.210
		259. kłobucki	1.042
		260. lubliniecki	1.022
		261. mikołowski	1.266
		262. myszkowski	1.232
		263. pszczyński	1.411
		264. raciborski	1.422
		265. rybnicki	1.731

		266. tarnogórski	1.324
		267. bieruńsko-lędziński	1.766
		268. wodzisławski	1.468
		269. zawierciański	0.859
		270. żywiecki	1.789
		271. c. Bielsko-Biała	2.894
		272. c. Bytom	1.021
		273. c. Chorzów	1.049
		274. c. Częstochowa	0.896
		275. c. Dąbrowa Górnicza	0.938
		276. c. Gliwice	1.018
		277. c. Jastrzębie-Zdrój	0.993
		278. c. Jaworzno	0.931
		279. c. Katowice	0.820
		280. c. Mysłowice	3.077
		281. c. Piekary Śląskie	0.752
		282. c. Ruda Śląska	0.719
		283. c. Rybnik	1.220
		284. c. Siemianowice Śląskie	0.769
		285. c. Sosnowiec	3.264
		286. c. Świętochłowice	1.422
		287. c. Tychy	0.950
		288. c. Zabrze	1.181
		289. c. Żory	0.940
13.	Świętokrzyskie	290. buski	1.103
		291. jędrzejowski	1.228
		292. kazimierski	1.102
		293. kielecki	1.668
		294. konecki	0.737
		295. opatowski	0.837

		296. ostrowiecki	1.024
		297. pińczowski	0.727
		298. sandomierski	0.835
		299. skarżyski	1.322
		300. starachowicki	0.598
		301. staszowski	0.717
		302. włoszczowski	1.191
		303. c. Kielce	0.840
14.	Warmia and Mazury	304. bartoszycki	0.698
		305. braniewski	0.720
		306. działdowski	1.878
		307. elbląski	0.876
		308. ełcki	0.283
		309. giżycki	0.643
		310. iławski	0.513
		311. kętrzyński	1.371
		312. lidzbarski	0.380
		313. mrągowski	0.627
		314. nidzicki	1.403
		315. nowomiejski	0.592
		316. olecki	0.229
		317. olsztyński	0.588
		318. ostródzki	0.303
		319. piski	0.336
		320. szczycieński	0.912
		321. gołdapski	0.259
		322. węgorzewski	0.558
		323. c. Elbląg	0.643
		324. c. Olsztyn	0.705

15.	Wielkopolska	325. chodzieski	0.842
		326. czarnkowsko-trzcianecki	0.599
		327. gnieźnieński	0.705
		328. gostyński	0.708
		329. grodziski	0.460
		330. jarociński	0.736
		331. kaliski	1.294
		332. kępiński	1.125
		333. kolski	1.179
		334. koniński	1.298
		335. kościański	0.391
		336. krotoszyński	0.782
		337. leszczyński	0.607
		338. międzychodzki	0.685
		339. nowotomyski	0.692
		340. obornicki	0.971
		341. ostrowski	0.582
		342. ostrzeszowski	1.521
		343. pilski	0.672
		344. pleszewski	0.835
		345. poznański	1.033
		346. rawicki	0.617
		347. słupecki	1.589
		348. szamotulski	0.867
		349. średzki	0.629
		350. śremski	0.711
		351. turecki	1.070
		352. wągrowiecki	0.843
		353. wolsztyński	0.777
		354. wrzesiński	0.643

		355. złotowski	0.852
		356. c. Kalisz	1.139
		357. c. Konin	1.055
		358. c. Leszno	0.788
		359. c. Poznań	1.037
16.	West Pomerania	360. białogardzki	0.865
		361. choszczeński	0.587
		362. drawski	0.946
		363. goleniowski	0.533
		364. gryficki	0.923
		365. gryfiński	0.642
		366. kamieński	0.800
		367. kołobrzeski	1.157
		368. koszaliński	0.846
		369. myśliborski	0.775
		370. policki	0.860
		371. pyrzycki	0.629
		372. sławieński	0.654
		373. stargardzki	0.731
		374. szczecinecki	0.913
		375. świdwiński	1.082
		376. wałecki	0.728
		377. łobeski	1.141
		378. c. Koszalin	0.852
		379. c. Szczecin	0.707
		380. c. Świnoujście	0.946

Source: own elaboration
