# Supplementary material for: The Evaluation of Municipal Waste in Counties in Poland with the Use of the Theory of Phenomena Spatial Concentration
Source: Int J Environ Res Public Health. 2020 Dec 6;17(23):9107. doi: 10.3390/ijerph17239107 (PMC7731399; doi:10.3390/ijerph17239107)
Supplement: Supplementary file 1 [file ijerph-17-09107-s001.zip › Supplementary materials/Tabele.S2.pdf]

Table S2. The values of the location quotients (*LQ*) for the analyzed fractions of selected waste in comparison with all selected waste in counties in Poland

No. voivodeship	Voivodeship	Counties	Location quotient (*LQ*)					
			Paper and cardboard (*PC*)	Glass (*G*)	Plastic (*P*)	Waste electrical and electronic equipment (*WEEE*)	Bulky waste (*BW*)	Biodegradable waste (*B*)
1.	Lower Silesia	1. bolesławiecki	0.745	1.167	0.441	1.071	0.882	1.566
		2. dzierżoniowski	1.720	1.388	0.996	0.629	2.253	0.555
		3. głogowski	1.396	0.956	0.796	0.541	1.494	1.048
		4. górowski	1.497	1.895	3.909	0.198	0.932	0.412
		5. jaworski	1.573	1.667	0.029	1.135	0.657	0.793
		6. jeleniogórski	1.120	1.124	2.106	0.293	1.161	1.332
		7. kamiennogórski	1.956	1.406	0.901	1.347	1.331	0.486
		8. kłodzki	1.040	1.266	0.532	1.130	1.450	0.950
		9. legnicki	0.399	0.777	0.652	0.364	0.567	1.385
		10. lubański	0.580	1.115	0.657	0.112	1.742	1.585
		11. lubiński	1.337	1.025	1.002	3.137	1.255	1.048
		12. lwówecki	0.823	1.753	0.848	0.272	0.955	0.771
		13. milicki	0.650	1.958	2.393	0.128	0.461	1.028
		14. oleśnicki	1.165	1.344	1.362	1.085	1.334	1.139
		15. oławski	1.124	1.694	0.212	1.816	1.302	1.215
		16. polkowicki	1.902	1.459	0.026	1.277	1.307	0.495
		17. strzeliński	0.889	2.047	0.003	0.671	0.485	1.281
		18. średzki	1.812	1.877	0.534	0.481	1.087	0.429
		19. świdnicki	2.055	1.416	1.873	0.826	1.141	0.616
		20. trzebnicki	1.007	1.072	2.096	0.337	0.990	1.498
		21. wałbrzyski	1.593	1.369	2.282	0.546	1.109	0.520
		22. wołowski	1.058	1.203	1.289	0.185	1.906	1.146

		23. wrocławski	1.084	1.189	1.494	0.520	0.693	1.354
		24. ząbkowicki	0.346	0.877	0.388	0.539	2.838	0.983
		25. zgorzelecki	1.075	1.004	0.509	0.432	1.830	1.455
		26. złotoryjski	3.600	1.403	1.053	0.030	0.671	0.359
		27. c. Jelenia Góra	2.191	1.675	0.103	1.072	0.930	0.286
		28. c. Legnica	0.786	0.999	0.671	0.128	0.960	0.810
		29. c. Wrocław	2.822	1.032	0.039	0.646	1.047	0.799
		30. c. Wałbrzych	1.886	0.084	0.609	8.687	3.411	0.606
2.	Kujawy and Pomerania	31. aleksandrowski	0.468	0.719	1.181	0.402	0.422	1.367
		32. brodnicki	0.518	0.970	2.334	0.391	0.673	1.477
		33. bydgoski	0.672	1.070	0.770	0.335	0.554	1.584
		34. chełmiński	0.513	1.503	0.126	1.529	0.864	1.227
		35. golubsko-dobrzyński	0.932	1.791	2.690	1.047	0.446	1.069
		36. grudziądzki	0.631	1.645	3.607	0.824	0.158	1.269
		37. inowrocławski	0.699	1.004	1.445	0.180	0.501	2.034
		38. lipnowski	0.012	0.572	2.007	1.202	0.602	0.927
		39. mogileński	0.586	1.056	1.576	0.517	0.618	1.209
		40. nakielski	1.655	1.808	2.428	1.550	0.935	0.644
		41. radziejowski	0.296	1.017	0.924	0.592	0.453	0.457
		42. rypiński	1.933	1.419	2.041	1.464	0.382	1.013
		43. sępoleński	0.152	1.448	0.819	0.950	0.583	0.925
		44. świecki	0.565	1.272	2.711	4.073	0.580	1.281
		45. toruński	0.237	0.919	1.049	0.679	0.353	1.815
		46. tucholski	0.491	1.239	1.999	1.504	0.597	0.804
		47. wąbrzeski	0.741	1.140	1.880	1.262	0.842	1.475
		48. włocławski	0.335	0.621	0.671	0.278	0.168	1.016
		49. żniński	1.219	1.794	3.507	1.228	0.732	0.770
		50. c. Bydgoszcz	0.229	1.245	0.102	1.111	1.129	1.246
		51. c. Grudziądz	1.696	1.004	2.301	0.059	0.005	1.847
		52. c. Toruń	1.734	1.130	1.637	0.499	1.030	1.392

		53. c. Włocławek	0.599	0.646	0.844	0.801	0.573	2.333
3.	Lublin	54. bialski	1.293	1.396	0.829	0.760	0.297	0.513
		55. biłgorajski	1.621	1.937	1.773	2.906	0.746	0.523
		56. chełmski	0.335	2.045	0.791	1.724	0.450	0.211
		57. hrubieszowski	0.617	2.074	0.811	2.753	0.991	0.360
		58. janowski	0.849	1.590	0.614	1.482	1.748	0.694
		59. krasnostawski	0.970	2.415	3.063	2.346	0.680	0.591
		60. kraśnicki	0.648	1.040	0.110	0.617	0.982	1.229
		61. lubartowski	0.580	1.385	1.876	2.036	0.643	1.145
		62. lubelski	0.916	1.447	2.009	2.496	1.067	0.684
		63. łęczyński	0.821	1.083	0.543	0.767	0.607	0.398
		64. łukowski	0.729	0.971	0.465	0.534	0.396	1.129
		65. opolski	1.095	1.602	2.441	2.436	0.825	0.365
		66. parczewski	0.376	1.374	0.109	1.579	0.411	1.133
		67. puławski	1.153	1.097	1.344	1.208	0.654	1.191
		68. radzyński	0.311	0.744	0.705	0.968	0.260	1.323
		69. rycki	1.398	1.826	0.546	1.318	0.447	0.751
		70. świdnicki	1.269	1.217	2.018	1.130	1.055	0.923
		71. tomaszowski	0.225	0.827	0.750	2.559	0.771	0.921
		72. włodawski	0.185	0.401	0.021	1.419	0.719	0.183
		73. zamojski	1.039	1.993	1.971	3.092	0.605	0.305
		74. c. Biała Podlaska	2.211	1.070	0.344	0.787	0.314	0.122
		75. c. Chełm	0.689	0.788	0.251	0.955	0.231	1.097
		76. c. Lublin	1.004	0.727	0.539	0.414	0.949	1.212
		77. c. Zamość	1.959	0.962	1.834	1.704	0.745	0.900
4.	Lubuskie	78. gorzowski	0.825	0.856	1.002	0.078	1.732	1.400
		79. krośnieński	0.800	1.211	2.788	0.950	1.833	0.842
		80. międzyrzecki	1.716	1.127	2.350	0.383	1.278	1.052
		81. nowosolski	0.825	0.794	1.542	0.037	1.015	1.867

		82. słubicki	1.074	0.651	1.614	0.880	1.821	1.430
		83. strzelecko-drezdenecki	0.888	0.862	1.573	0.088	2.146	0.941
		84. sulęciński	1.725	0.872	1.267	0.228	2.027	1.188
		85. świebodziński	1.551	0.969	2.750	0.030	1.090	0.691
		86. zielonogórski	0.858	0.819	2.076	0.310	1.852	1.038
		87. żagański	1.583	1.500	1.559	1.107	1.813	0.822
		88. żarski	1.198	0.961	1.417	0.123	1.544	1.273
		89. wschowski	0.168	1.241	3.214	1.071	1.063	1.255
		90. c. Gorzów Wielkopolski	1.268	0.498	1.102	0.573	1.457	1.783
		91. c. Zielona Góra	2.653	0.952	1.959	0.568	1.254	0.832
5.	Łódź	92. bełchatowski	0.743	1.193	0.534	0.126	1.120	1.090
		93. kutnowski	0.070	0.722	0.424	0.332	0.270	1.772
		94. łaski	0.107	1.121	0.209	0.268	0.893	1.084
		95. łęczycki	0.191	1.395	0.899	1.021	0.978	0.268
		96. łowicki	0.295	1.643	0.114	1.945	0.836	0.847
		97. łódzki wschodni	2.222	1.743	1.348	0.532	0.553	0.878
		98. opoczyński	3.099	2.277	2.031	0.860	0.798	0.097
		99. pabianicki	0.117	0.806	0.164	0.358	2.115	1.156
		100. pajęczański	0.380	2.076	1.208	0.562	1.143	0.272
		101. piotrkowski	1.071	1.522	1.264	0.334	0.831	0.702
		102. poddębicki	0.421	1.264	0.634	0.746	1.096	1.035
		103. radomszczański	0.716	1.012	1.442	0.722	0.801	1.098
		104. rawski	3.155	1.434	2.537	1.189	0.967	0.485
		105. sieradzki	1.235	1.460	0.875	1.683	0.904	0.630
		106. skierniewicki	0.082	1.923	0.379	0.091	1.515	0.153
		107. tomaszowski	1.854	1.476	1.967	0.492	1.369	0.726
		108. wieluński	0.325	1.861	0.019	0.100	1.459	0.436
		109. wieruszowski	0.000	1.759	0.001	0.003	1.873	0.356
		110. zduńskowolski	1.361	1.006	0.959	0.039	0.057	0.012
		111. zgierski	0.591	0.801	0.905	1.012	0.733	0.747

		112. brzeziński	0.374	0.573	0.680	0.048	2.667	0.640
		113. c. Łódź	0.129	0.171	0.398	1.088	0.806	1.886
		114. c. Piotrków Trybunalski	3.004	2.080	2.166	0.849	0.322	0.742
		115. c. Skierniewice	0.635	0.967	0.015	0.239	0.467	1.771
6.	Małopolska	116. bocheński	1.195	1.632	2.430	0.915	1.058	0.911
		117. brzeski	0.870	1.719	2.704	1.828	1.654	0.064
		118. chrzanowski	0.984	1.320	2.088	0.703	1.197	1.185
		119. dąbrowski	1.124	1.973	4.041	1.763	1.121	0.189
		120. gorlicki	1.377	1.309	3.035	0.368	1.679	0.344
		121. krakowski	1.667	0.990	1.919	1.130	1.631	0.578
		122. limanowski	0.417	1.257	3.010	1.046	1.804	0.358
		123. miechowski	1.000	1.877	2.844	2.101	0.581	0.258
		124. myślenicki	0.436	0.924	0.872	0.411	1.281	1.108
		125. nowosądecki	0.893	1.723	2.887	0.805	1.725	0.436
		126. nowotarski	0.298	0.952	1.800	0.411	1.613	0.558
		127. olkuski	0.976	1.310	0.810	3.316	1.089	0.958
		128. oświęcimski	0.806	1.353	1.001	1.251	1.404	1.088
		129. proszowicki	0.998	2.923	1.576	3.616	0.892	0.135
		130. suski	0.464	1.432	3.474	0.941	1.771	0.263
		131. tarnowski	1.004	1.589	2.653	0.988	1.505	0.519
		132. tatrzański	0.892	1.250	0.754	0.830	1.755	0.507
		133. wadowicki	0.625	1.631	2.806	0.557	1.539	0.580
		134. wielicki	0.855	0.981	1.240	0.388	1.461	0.705
		135. c. Kraków	0.178	0.548	0.120	0.716	0.802	1.293
		136. c. Nowy Sącz	1.740	1.147	2.289	0.237	1.974	0.741
		137. c. Tarnów	1.028	0.391	1.906	0.046	1.782	1.454
7.	Mazovia	138. białobrzeski	1.509	1.111	1.205	0.415	0.736	0.076
		139. ciechanowski	0.951	1.080	1.171	0.942	0.599	1.570
		140. garwoliński	4.416	1.310	1.760	2.601	0.559	0.516

		141. gostyniński	0.040	1.130	1.059	0.912	0.698	0.786
		142. grodziski	1.081	0.698	0.514	0.433	0.856	1.068
		143. grójecki	4.572	0.822	0.877	18.059	0.529	0.281
		144. kozienicki	1.436	1.536	1.128	0.416	0.898	1.006
		145. legionowski	0.544	0.615	0.220	3.531	1.027	0.965
		146. lipski	0.828	2.265	0.510	0.992	0.867	0.116
		147. łosicki	0.624	1.979	1.679	1.604	0.391	0.742
		148. makowski	0.899	1.217	1.073	0.686	0.573	0.579
		149. miński	1.425	1.331	1.846	0.859	0.688	0.484
		150. mławski	0.080	0.125	0.140	0.590	1.899	0.576
		151. nowodworski	1.421	0.936	1.044	1.567	0.905	0.326
		152. ostrołęcki	2.844	1.141	2.366	4.698	0.885	0.331
		153. ostrowski	2.084	1.171	1.530	2.783	0.457	0.934
		154. otwocki	2.571	0.764	1.061	0.624	0.592	1.474
		155. piaseczyński	0.948	0.773	0.396	0.609	0.753	1.161
		156. płocki	0.342	1.365	0.679	1.304	0.833	0.658
		157. płoński	1.663	1.358	2.229	1.916	1.552	0.568
		158. pruszkowski	2.468	0.822	1.271	0.464	1.056	0.856
		159. przasnyski	2.116	0.730	0.926	4.271	0.500	0.667
		160. przysuski	1.079	2.588	3.517	1.956	0.681	0.000
		161. pułtuski	1.957	1.196	1.877	0.392	0.105	0.138
		162. radomski	1.036	1.409	1.379	0.475	0.844	0.587
		163. siedlecki	0.246	1.496	3.102	0.982	0.453	0.547
		164. sierpecki	0.779	1.689	1.641	0.701	0.357	1.466
		165. sochaczewski	0.774	1.369	0.632	0.068	1.252	0.673
		166. sokołowski	1.611	1.722	2.531	1.961	0.444	0.228
		167. szydłowiecki	0.655	1.985	1.418	0.584	0.595	0.482
		168. warszawski zachodni	1.028	0.427	0.460	0.831	1.010	1.091
		169. węgrowski	0.328	1.416	3.286	3.292	0.640	0.387
		170. wołomiński	1.607	0.952	1.667	1.095	0.563	1.567

		171. wyszkowski	3.060	1.256	2.223	0.217	0.934	0.430
		172. zwoleński	1.094	1.635	1.664	1.002	0.712	0.671
		173. żuromiński	0.288	2.007	1.462	1.790	0.947	0.559
		174. żyrardowski	0.648	1.418	0.430	0.402	0.648	0.221
		175. c. Ostrołęka	2.115	0.573	1.836	0.598	0.753	0.930
		176. c. Płock	0.324	0.654	0.012	0.493	1.517	1.074
		177. c. Radom	0.810	0.637	0.158	0.001	0.744	0.831
		178. c. Siedlce	1.623	0.760	1.851	0.400	0.611	1.409
		179. c. Warszawa	0.054	0.593	0.043	3.190	1.380	0.719
8.	Opole	180. brzeski	0.357	1.078	0.003	0.422	0.843	1.109
		181. głubczycki	0.717	0.668	0.523	0.780	0.590	0.705
		182. kędzierzyńsko-kozielski	0.169	0.779	0.003	0.111	1.274	1.472
		183. kluczborski	0.146	1.496	1.456	0.510	0.719	0.720
		184. krapkowicki	0.083	0.578	0.000	0.038	1.171	1.578
		185. namysłowski	1.330	2.244	3.057	0.000	1.310	0.329
		186. nyski	0.420	1.322	0.863	0.748	0.865	0.225
		187. oleski	0.381	1.513	0.592	0.168	1.518	0.869
		188. opolski	0.627	1.170	1.319	0.566	0.996	0.954
		189. prudnicki	0.431	0.917	1.084	0.537	0.774	1.256
		190. strzelecki	0.110	0.777	0.211	0.788	0.931	1.293
		191. c. Opole	0.069	0.748	0.041	0.318	0.988	1.297
9.	Podkarpacie	192. bieszczadzki	0.533	2.341	2.286	4.119	2.268	0.153
		193. brzozowski	0.581	1.286	0.468	0.569	1.822	0.140
		194. dębicki	0.833	1.319	0.997	1.751	0.969	0.665
		195. jarosławski	2.495	1.142	1.039	0.666	1.437	0.561
		196. jasielski	1.589	1.107	0.715	0.365	1.344	0.648
		197. kolbuszowski	0.733	2.915	2.501	1.138	0.847	0.188
		198. krośnieński	0.429	1.522	0.120	1.041	1.476	0.542
		199. leżajski	0.927	1.600	0.548	1.732	0.711	0.820

		200. lubaczowski	1.939	1.589	2.965	2.253	1.507	0.099
		201. łańcucki	0.664	2.252	1.057	2.012	0.719	0.567
		202. mielecki	1.213	1.592	1.449	0.705	1.430	0.811
		203. niżański	0.313	1.475	2.678	1.118	0.722	1.025
		204. przemyski	0.529	2.284	2.310	1.390	1.286	0.109
		205. przeworski	1.634	1.558	0.581	1.304	0.973	0.411
		206. ropczycko-sędziszowski	0.970	2.474	2.983	2.062	0.935	0.047
		207. rzeszowski	0.339	1.501	0.979	1.832	0.969	0.416
		208. sanocki	1.421	1.395	0.610	2.603	1.546	0.813
		209. stalowowolski	1.273	1.320	2.347	1.110	1.378	0.700
		210. strzyżowski	0.410	1.331	0.782	0.078	1.342	0.298
		211. tarnobrzeski	1.794	1.725	3.338	0.662	1.299	0.266
		212. leski	0.472	2.698	3.930	2.487	1.166	0.092
		213. c. Krosno	0.072	1.271	0.033	0.284	1.850	0.877
		214. c. Przemyśl	2.869	1.553	0.000	1.772	1.008	0.340
		215. c. Rzeszów	0.298	0.535	0.161	0.356	0.522	0.486
		216. c. Tarnobrzeg	1.886	1.288	3.725	0.960	1.458	0.348
10.	Podlasie	217. augustowski	1.613	1.488	2.252	2.004	0.882	0.652
		218. białostocki	0.838	0.717	0.979	0.741	1.062	0.998
		219. bielski	0.374	0.552	0.256	1.023	0.505	1.687
		220. grajewski	1.418	1.082	2.037	2.943	0.675	0.959
		221. hajnowski	1.010	1.196	0.230	2.049	0.622	0.965
		222. kolneński	0.599	2.240	4.547	2.907	0.755	0.253
		223. łomżyński	0.442	1.877	3.150	1.440	1.617	0.158
		224. moniecki	0.565	1.058	1.874	3.475	0.774	0.595
		225. sejneński	0.446	2.384	0.309	3.416	0.409	0.055
		226. siemiatycki	1.354	1.733	0.250	1.495	0.464	0.724
		227. sokólski	1.242	1.834	1.935	1.977	1.044	0.302
		228. suwalski	2.144	2.115	1.426	2.689	0.720	0.093
		229. wysokomazowiecki	1.366	1.503	1.203	2.497	1.099	0.833

		230. zambrowski	1.302	1.033	0.877	2.620	1.683	0.839
		231. c. Białystok	0.888	0.837	0.423	0.609	1.264	1.051
		232. c. Łomża	1.994	1.009	2.074	0.949	0.995	1.140
		233. c. Suwałki	1.019	1.007	0.003	0.451	1.238	0.916
11.	Pomerania	234. bytowski	0.920	1.687	3.985	1.650	1.040	0.037
		235. chojnicki	0.050	1.158	0.141	1.189	0.806	0.055
		236. człuchowski	0.454	1.138	0.343	1.409	0.785	0.802
		237. gdański	1.388	1.007	0.966	0.861	0.567	1.731
		238. kartuski	0.475	1.167	0.442	1.034	0.909	1.204
		239. kościerski	0.221	0.368	0.193	1.183	0.412	1.120
		240. kwidzyński	1.168	1.306	2.593	0.955	0.705	0.908
		241. lęborski	0.211	1.013	0.114	0.476	0.846	1.017
		242. malborski	0.874	1.159	2.541	0.446	0.472	1.194
		243. nowodworski	1.245	1.735	3.073	2.210	0.758	0.812
		244. pucki	1.002	1.027	0.502	0.664	0.901	1.239
		245. słupski	0.898	1.302	2.553	1.256	1.161	0.854
		246. starogardzki	0.212	0.390	0.051	2.397	0.329	1.636
		247. tczewski	0.859	1.301	2.967	1.037	0.843	0.803
		248. wejherowski	1.474	1.221	2.085	1.211	0.687	1.146
		249. sztumski	1.138	1.274	2.173	1.268	0.387	1.380
		250. c. Gdańsk	1.369	0.838	0.053	0.170	0.827	2.009
		251. c. Gdynia	2.769	1.318	2.249	0.974	1.128	0.779
		252. c. Słupsk	1.433	1.043	3.104	1.911	1.103	0.741
		253. c. Sopot	2.622	1.420	1.537	1.700	1.050	0.625
12.	Silesia	254. będziński	0.903	0.925	0.769	0.243	1.398	1.319
		255. bielski	0.246	0.728	0.561	0.772	0.654	0.561
		256. cieszyński	0.988	0.797	1.390	0.691	0.908	0.483
		257. częstochowski	0.348	1.414	1.833	0.901	0.441	0.593
		258. gliwicki	1.035	0.858	0.870	0.325	1.452	1.423

		259. kłobucki	0.417	1.436	1.874	1.133	0.601	0.566
		260. lubliniecki	0.433	1.369	2.072	0.681	0.656	0.953
		261. mikołowski	0.990	0.934	0.492	0.243	1.341	1.176
		262. myszkowski	0.659	1.042	1.750	0.785	0.520	1.003
		263. pszczyński	1.027	0.811	1.073	0.534	0.999	0.376
		264. raciborski	0.493	0.760	0.225	0.704	1.210	0.668
		265. rybnicki	0.350	0.651	0.410	0.270	0.819	1.206
		266. tarnogórski	0.815	0.910	1.228	0.092	1.923	1.154
		267. bieruńsko-lędziński	0.736	0.552	1.001	0.494	0.947	0.948
		268. wodzisławski	0.357	0.678	0.562	0.536	1.136	0.623
		269. zawierciański	1.006	1.645	2.488	0.354	0.581	1.106
		270. żywiecki	0.190	1.253	0.803	1.271	0.795	0.696
		271. c. Bielsko-Biała	0.100	0.461	0.019	2.861	0.412	1.586
		272. c. Bytom	1.955	0.947	1.016	0.095	1.116	0.407
		273. c. Chorzów	0.955	0.705	0.000	0.181	1.627	1.728
		274. c. Częstochowa	0.805	0.573	0.953	1.279	0.551	2.008
		275. c. Dąbrowa Górnicza	1.294	1.104	0.000	0.000	0.860	1.537
		276. c. Gliwice	2.522	0.712	1.757	0.212	1.795	0.822
		277. c. Jastrzębie-Zdrój	0.930	0.963	1.733	0.698	1.603	1.082
		278. c. Jaworzno	1.018	0.860	1.175	0.415	1.134	1.868
		279. c. Katowice	1.784	0.732	0.474	0.430	1.967	0.800
		280. c. Mysłowice	0.274	0.270	0.044	0.151	0.458	0.389
		281. c. Piekary Śląskie	0.674	0.967	1.391	0.818	1.805	1.379
		282. c. Ruda Śląska	0.842	0.826	1.209	0.136	1.649	1.661
		283. c. Rybnik	0.932	0.695	1.475	0.252	0.902	1.041
		284. c. Siemianowice Śląskie	1.940	1.198	1.829	0.151	2.142	0.730
		285. c. Sosnowiec	0.009	0.281	0.007	0.045	0.417	0.350
		286. c. Świętochłowice	0.271	0.430	0.373	0.019	0.785	0.786
		287. c. Tychy	2.016	1.124	1.976	0.010	0.961	1.085
		288. c. Zabrze	1.870	1.178	2.534	0.062	1.065	1.089

		289. c. Żory	1.608	0.979	0.102	1.252	0.949	1.340
13.	Świętokrzyskie	290. buski	0.654	2.040	1.121	1.716	1.146	0.223
		291. jędrzejowski	1.488	2.146	1.679	1.327	0.526	0.119
		292. kazimierski	0.018	1.998	0.165	0.509	1.628	0.013
		293. kielecki	0.829	2.006	1.815	1.257	0.581	0.348
		294. konecki	0.593	1.641	0.332	1.822	0.822	0.759
		295. opatowski	0.852	0.533	1.445	1.168	0.724	0.026
		296. ostrowiecki	2.285	0.398	0.643	0.682	1.129	0.215
		297. pińczowski	0.322	1.879	0.964	1.434	0.981	0.005
		298. sandomierski	1.530	0.563	0.780	0.667	0.471	0.097
		299. skarżyski	0.399	0.516	1.726	3.328	1.218	0.093
		300. starachowicki	1.217	1.527	2.289	0.510	0.509	0.049
		301. staszowski	0.484	1.296	0.300	1.131	1.541	0.590
		302. włoszczowski	1.411	2.001	2.850	2.085	0.323	0.312
		303. c. Kielce	0.755	1.048	0.098	1.339	1.187	0.953
14.	Warmia and Mazury	3014. bartoszycki	2.811	1.420	2.530	1.473	0.520	0.901
		305. braniewski	1.436	1.238	2.865	1.672	1.021	0.418
		306. działdowski	0.597	0.753	1.119	0.426	0.275	1.069
		307. elbląski	0.998	1.309	2.794	0.854	0.680	0.698
		308. ełcki	1.473	0.048	0.030	1.710	2.864	0.633
		309. giżycki	1.498	0.935	0.058	1.524	0.874	0.956
		310. iławski	1.111	1.512	1.225	0.859	0.795	1.245
		311. kętrzyński	0.725	0.649	1.409	0.647	0.301	1.499
		312. lidzbarski	2.920	1.933	3.459	3.305	0.298	0.390
		313. mrągowski	1.801	1.325	2.087	1.864	0.780	0.651
		314. nidzicki	0.680	1.075	1.350	0.811	0.547	0.891
		315. nowomiejski	0.275	1.153	0.753	2.215	0.844	0.861
		316. olecki	0.021	0.039	0.011	3.799	2.923	0.421
		317. olsztyński	1.759	1.773	2.617	1.986	1.247	0.280

		318. ostródzki	0.455	1.164	0.812	1.166	1.771	0.351
		319. piski	1.706	1.435	1.283	3.226	1.814	0.244
		320. szczycieński	1.235	1.632	2.396	1.895	0.747	0.676
		321. gołdapski	0.228	0.300	0.065	2.747	2.035	0.309
		322. węgorzewski	0.840	1.186	0.001	2.776	0.846	0.841
		323. c. Elbląg	1.739	0.623	1.373	0.288	1.281	1.633
		324. c. Olsztyn	2.345	0.948	1.559	0.697	1.143	1.184
15.	Wielkopolska	325. chodzieski	1.569	1.648	3.107	0.314	0.602	0.976
		326. czarnkowsko-trzcianecki	0.395	1.433	1.333	2.039	1.648	0.900
		327. gnieźnieński	0.441	1.153	0.712	0.911	0.906	1.421
		328. gostyński	0.508	1.979	2.268	1.803	1.082	1.064
		329. grodziski	0.667	2.718	3.362	0.997	1.233	0.251
		330. jarociński	1.033	1.982	1.596	1.901	0.497	1.310
		331. kaliski	0.089	1.558	0.124	1.173	0.531	0.357
		332. kępiński	0.000	1.599	0.000	0.001	1.300	0.167
		333. kolski	0.755	1.303	1.492	0.766	0.458	1.160
		334. koniński	1.462	1.204	2.146	0.889	0.333	0.989
		335. kościański	0.768	2.490	2.722	0.579	1.019	0.654
		336. krotoszyński	0.522	1.389	2.338	1.045	0.498	1.609
		337. leszczyński	0.893	2.069	2.831	3.507	1.152	0.636
		338. międzychodzki	1.266	1.322	2.792	0.356	1.806	0.703
		339. nowotomyski	0.823	1.717	2.562	0.651	1.152	1.014
		340. obornicki	0.703	1.488	1.822	0.981	0.769	1.571
		341. ostrowski	1.100	1.774	1.736	1.343	1.132	0.434
		342. ostrzeszowski	0.275	1.167	0.069	0.387	1.094	1.584
		343. pilski	1.315	1.026	2.254	0.226	1.288	0.901
		344. pleszewski	0.858	1.527	2.247	2.087	1.573	0.377
		345. poznański	2.048	1.041	1.726	0.415	0.634	1.502
		346. rawicki	1.323	1.892	3.070	2.825	0.908	0.688
		347. słupecki	0.556	1.321	1.934	0.868	0.450	1.307

		348. szamotulski	1.096	1.439	2.527	0.256	0.664	1.346
		349. średzki	0.961	0.830	1.152	0.741	0.868	0.746
		350. śremski	1.418	1.196	1.862	0.498	0.789	1.541
		351. turecki	0.391	1.080	0.956	0.804	0.909	0.529
		352. wągrowiecki	0.927	1.518	2.721	0.875	0.654	1.282
		353. wolsztyński	0.780	1.413	3.834	1.169	1.080	0.779
		354. wrzesiński	1.450	1.220	2.124	0.348	0.630	1.473
		355. złotowski	0.377	0.699	1.790	0.612	2.062	0.497
		356. c. Kalisz	1.328	0.715	0.418	0.740	0.967	0.815
		357. c. Konin	1.319	0.799	1.599	0.020	0.389	2.080
		358. c. Leszno	1.273	1.171	1.675	1.614	0.933	1.527
		359. c. Poznań	2.188	1.327	1.309	1.420	1.070	1.255
16.	West Pomerania	360. białogardzki	2.023	1.295	1.324	0.918	1.084	0.869
		361. choszczeński	0.571	1.259	1.319	0.954	2.503	0.280
		362. drawski	0.499	0.951	1.023	0.048	1.624	0.837
		363. goleniowski	0.744	1.282	2.243	0.810	1.370	1.249
		364. gryficki	0.849	1.008	1.605	1.273	1.187	1.230
		365. gryfiński	1.219	1.357	0.109	0.367	2.060	0.681
		366. kamieński	1.069	1.154	1.642	1.469	1.321	1.135
		367. kołobrzeski	0.451	0.595	0.166	0.747	0.687	1.423
		368. koszaliński	0.699	1.576	0.162	1.057	1.141	0.943
		369. myśliborski	0.957	1.117	0.491	0.896	1.406	1.293
		370. policki	2.033	0.926	0.826	1.136	1.130	1.462
		371. pyrzycki	0.772	1.611	0.011	1.309	1.424	0.519
		372. sławieński	1.057	1.637	0.426	3.956	1.017	1.261
		373. stargardzki	1.449	1.297	1.068	0.838	1.235	1.079
		374. szczecinecki	1.351	1.340	0.087	0.867	1.172	0.900
		375. świdwiński	0.474	1.148	1.442	0.169	1.355	0.810
		376. wałecki	1.392	1.229	1.186	0.422	1.660	0.582
		377. łobeski	0.320	1.175	1.749	0.681	1.040	1.268

		378. c. Koszalin	2.293	1.086	0.308	1.127	0.493	1.392
		379. c. Szczecin	3.369	1.177	0.595	1.418	1.303	0.912
		380. c. Świnoujście	0.618	0.676	0.012	1.291	2.354	0.807

Source: own elaboration
